# Vanishing river ice cover in the lower part of the Danube basin – signs of a changing climate

**DOI:** 10.1038/s41598-018-26357-w

**Published:** 2018-05-21

**Authors:** M. Ionita, C. -A. Badaluta, P. Scholz, S. Chelcea

**Affiliations:** 1 Alfred Wegener Institute, Helmholtz Center for Polar and Marine Research, Bremerhaven, Germany; 2 Stable Isotope Laboratory, Ștefan cel Mare University, Suceava, Romania; 3 Department of Geography, Stefan cel Mare University, Suceava, Romania; 4 National Institute of Hydrology and Water Management, Bucharest, Romania

## Abstract

Many of the world’s largest rivers in the extra tropics are covered with ice during the cold season, and in the Northern Hemisphere approximately 60% of the rivers experience significant seasonal effects of river ice. Here we present an observational data set of the ice cover regime for the lower part of the Danube River which spans over the period 1837–2016, and its the longest one on record over this area. The results in this study emphasize the strong impact of climate change on the occurrence of ice regime especially in the second part of the 20^th^ century. The number of ice cover days has decreased considerably (~28days/century) mainly due to an increase in the winter mean temperature. In a long-term context, based on documentary evidences, we show that the ice cover occurrence rate was relatively small throughout the Medieval Warm Period (MWP), while the highest occurrence rates were found during the Maunder Minimum and Dalton Minimum periods. We conclude that the river ice regime can be used as a proxy for the winter temperature over the analyzed region and as an indicator of climate-change related impacts.

## Introduction

The formation of ice on lakes and rivers is a complex phenomenon that involves many meteorological, hydrological and physiographical properties of the catchment area. The meteorological and hydrological factors tend to vary both in time in space, the same being true for the ice conditions. Linking the occurrence of ice on lake and rives to climatic forcing might be a complex task to fulfill, however this task is simplified considerably by the fact that air temperature is the dominant factor driving the ice phenology^1–3^. As such, changes in river ice cover can be seen as an indication of changes in the climate forcing factors (e.g. air temperature, relative humidity, snowfall). As the global warming is anticipated to continue^4^, the continuous monitoring of lake and river ice could provide an early indicator of predicted global and regional warming. Due to the fact that ice record integrates climatic conditions during the late autumn/winter/spring months, when most of the warming is observed^5^, long-term observation of river ice can be extremely valuable. Long-term series of ice phenology have been employed successfully as indicators of past regional climate in Japan^6^, Finland^7,8^, Switzerland^9,10^, Canada^1,11–13^, Hungary^14,15^, as well as at hemispheric level^16^.

At European level, lake and river ice cover has been associated with local weather at seasonal scales, such as temperature and precipitation, and large-scale teleconnection patterns, like the North Atlantic Oscillation and El Nino-Southern Oscillation^17,18^. In addition to the natural variability, anthropogenic influences may also contribute to changes in the lake and river ice phenology, by alternating the large-scale atmospheric circulation, or alternations in land-use and human development^16^. Human interference in the river basin can lead to significant changes in ice conditions, especially by regulating the river flow and the construction of hydro-power stations.

**Figure 1:**
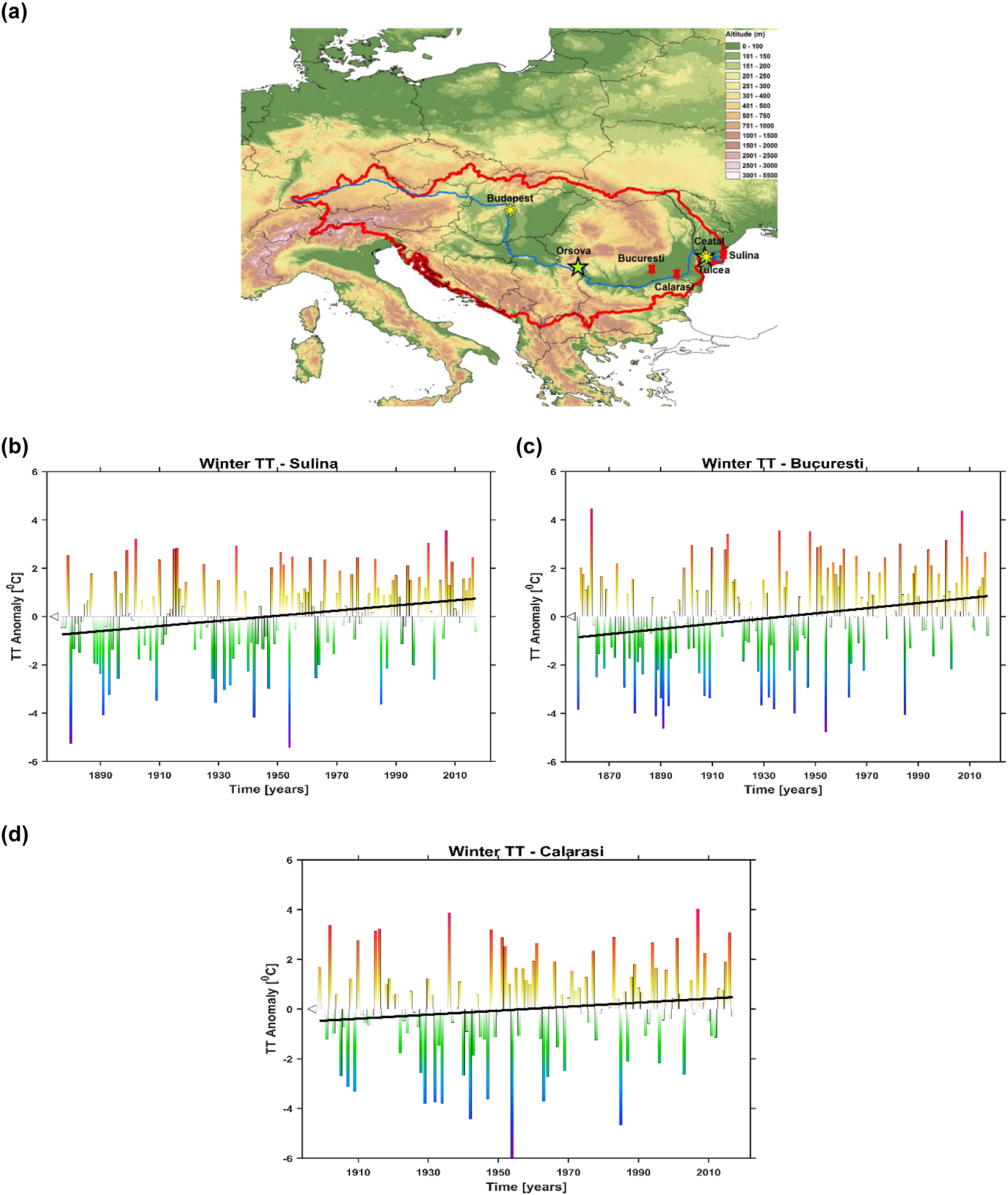
(**a**) Location of the Danube River catchment area and the hydrological (green stars), meteorological (red pins) and the ice cover stations (yellow star) analyzed in this study; (**b**) the time series of the winter (DJF) mean air temperature at Sulina station and its corresponding trend (magenta line) over the period 1855–2013; (**c**) the time series of the winter (DJF) mean air temperature at Bucuresti station and its corresponding trend (magenta line) over the period 1898–2013 and (**d**) the time series of the winter (DJF) mean air temperature at Calarasi station and its corresponding trend (magenta line) over the period 1875–2013. The identified trend is significant at the 99% significance level at the Bucuresti and Sulina station and not significant at Calarasi station (see Table S1).

In order to be able to study the variability of river ice cover duration, here we will show a long-term record of the duration of ice cover over the lower part of the Danube river basin (in the near vicinity of the Danube Delta). This is the first long-term record of ice cover duration over this area, which extends back ~180 years. The river ice record was collected starting 1836 by the Danube River Commission^19^. The record has been collected just over the lower part of the Danube River at Tulcea station (Fig. 1a). The ice regime of rivers and lakes can be characterized by the dates of appearance (freeze-up) and disappearance (break-up), as well as by the duration (ice cover) and frequency of the different ice phenomena during the winter season^20^. At the Tulcea station, we have a continuous record of the freeze-up date, break-up date and ice cover duration. Based on an extended compilation of documentary evidences we have reconstructed the occurrence of ice covered winters, in the lower part of Danube River, starting from ~850 AD up to 1830’s.

## Local Climate

The Danube River Basin is the second largest river basin in Europe, after the Volga River, covering an area of 801 463 km^2^. In the upper course, the Danube regime is determined by its alpine tributaries. The middle and lower course of the river stand under the influence of Drava and Sava rivers. Because of its large extension from west to east, and diverse topography (Fig. 1a), the Danube River Basin also shows large climatic differences. The upper region, in the west, features strong influence from the Atlantic climate with high precipitation, whereas the eastern regions are affected by continental climate with lower precipitation and typical cold winters. The precipitation ranges from <500 mm/year in the areas located at lower altitudes of up to >2000mm in the areas located at higher altitudes^21^. The highest annual temperature average with values of +12 °C is recorded in the middle and lower parts of Danube River Basin, while the coldest regions are at the heights of the mountains (e.g. the Carpathians and the Alps)^22^.

The temperature regime in the lower part of the Danube River, close to the Danube Delta, can be described by the data recorded at Sulina, Bucuresti and Calarasi meteorological stations. We choose these particular stations due to their location in the near proximity of our area of interest and their lengthy time span: Sulina (1876–2013, Fig. 1b), Bucuresti (1857–2013, Fig. 1c) and Calarasi (1898–2013, Fig. 1d). Increasing mean winter temperatures were observed at all three analyzed stations. The fastest winter warming was detected at Sulina station (Fig. 1b, Table S1), where the mean winter temperature growth rate was 1.06 °C/100 years (99% significance level). Sulina meteorological station is the station closest to the location of the ice cover data. The growth rate of the winter mean temperature at Bucuresti station (Fig. 1b) was 1.01 °C/100 years (99% significance level, Table S1) and 0.80 °C/100 years at Calarasi station (not significant, Fig. 1d, Table S1). The mean monthly temperature trends for the whole ice affected season (December–March) were also analyzed (not shown). The month with the highest growth rate in the mean temperature was the month of January, at all analyzed stations, coinciding with the main period of river ice formation.

## Ice cover Variability and Trends

Ice occurrence in the lower part of the Danube River depends strongly on the prevailing air temperature and large-scale atmospheric circulation^23^. Ice can occur over the whole Romanian part of the Danube main course, due to intrusions of cold air masses and low variability in the mean winter discharge. Overall, the main drivers of the river ice regime, in the lower part of the Danube catchment area are: air temperature and wind (meteorological factors); depth, slope and water speed (hydraulic factors) and discharge (hydrological factor). Due to its location, the Danube catchment area is under the influence of different air masses, which present a high interannual variability. Hence, the ice regime presents an increased variability in the freeze-up date, break-up date and ice cover duration throughout its catchment area. Over the Romanian part, between Orsova and Cetate gauging stations (Figure S1), due to a high slope and increase water speed, the formation of winter ice regime occurs just in extremely exceptional cases. Due to different meteorological conditions (winters are colder downstream) and hydro-geomorphological factors, downstream of Calarasi gauging station the occurrence of ice regime is more frequent compared to the regions upstream. The ice regime occurs downstream of Calarasi gauging station between December and March. On the basis of observations the earliest freeze-up date was 6^th^ December 1902 and the latest break-up date was 29^th^ March 1929.

**Figure 2:**
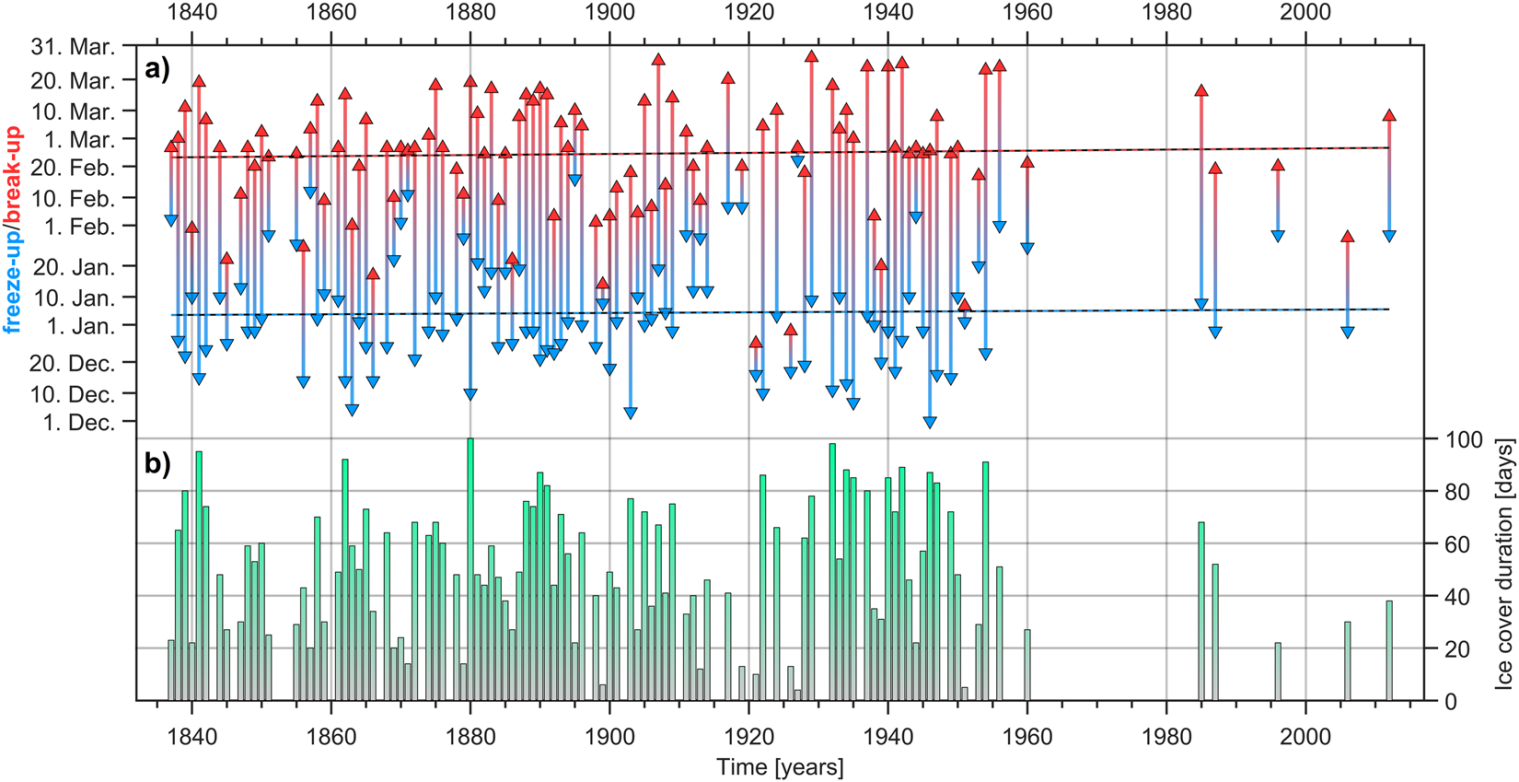
(**a**) The date of freeze-up and break-up and (**b**) the ice cover duration (days/winter) at Tulcea station, in the lower part of the Danube basin. Blue arrows indicate the freeze-up dates and red arrows indicate the break-up dates.

The first observation for the ice cover duration were started in 1836, at Tulcea station, by the Danube Commission^19^. These observations were continued throughout time, and based on a compilation from different sources^23–25^, here we present the first long-term record of ice cover regime in the lower part of Danube River (in the near vicinity of Danube Delta) (Fig. 2). On the lower part of the Danube the occurrence dates of ice phenomena varied over a wide time range. River ice cover usually occurred between the beginning of December and end of March (Fig. 2a). Over the investigated section of the Danube main course (the Romanian part), the length of the ice affected season over the period 1837–1950 is ~32 days/winter^22^. The longest ice cover period was recorded throughout the winter 1879/80, when the ice cover persisted for 101 days (Fig. 2b). One of the most striking feature of this ice cover record is the abrupt shift at ~1950 towards an almost ice free regime. Over the period 1837–1950, ice cover was present almost every year, with some small exceptions. Over the period 1951–2016, there were just ten winters when ice cover occurred and the number of days with ice covered is much smaller compared to the period 1837–1950. Although we identified significant changes in the ice cover duration, no significant changes were observed regarding the freeze-up and break-up dates (Fig. 2a).

**Figure 3:**
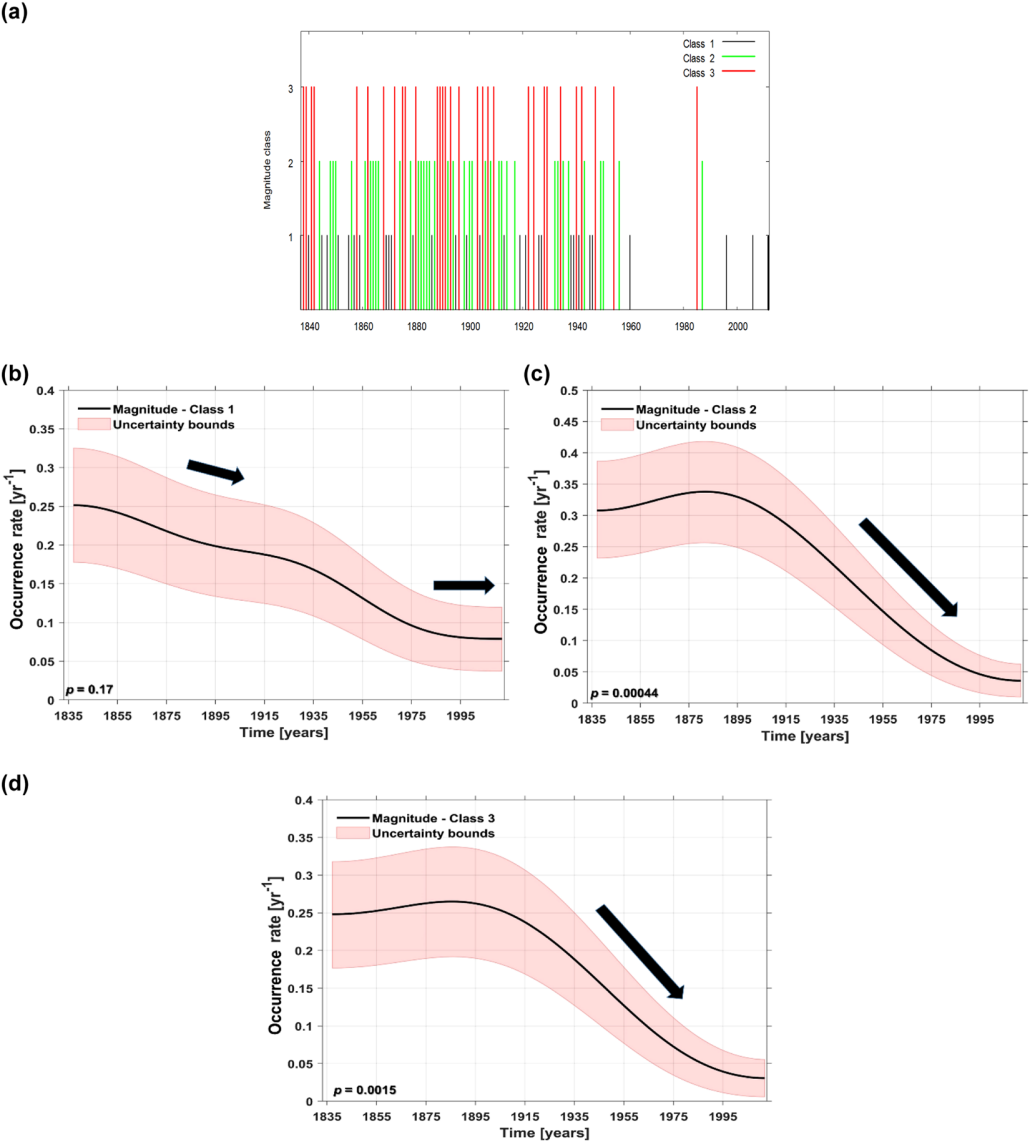
(**a**) Ice cover magnitude at Tulcea: black bars (class 1) indicate the years with ice cover between 1 and 60 days/winter, green bars (class 2) indicate the years with ice cover between 61 and 90 days/winter and red bars (class 3) indicate the years with ice cover >90 days/winter; (**b**) Occurrence of ice cover from class 1; (**c**) occurrence of ice cover from class 2; (**d**) occurrence of ice cover from class 3. (**a**) was analyzed using a Gaussian kernel, a bandwidth of 35 years and bootstrap simulations (see Methods). The black lines in (**b**,**c**) and (**d**) indicate the occurrence rate and the magenta shaded areas indicate the 90% confidence bands. The black arrows indicate the sign of the trend (downward for all classes). The trend is significant for class 2 and class 3 and not significant for class 1.

To test the possible trends, in the occurrence rates and the years of change in the mean, we have applied different statistical tests (see Methods) to our ice cover time series. First we have separated the time series in three different magnitude classes: a) class 1 (1 ≤ ice cover duration ≤60 days); b) class 2 (60 < ice cover duration ≤90 days) and c) class 3 (ice cover duration >90 days). Figure 3a illustrates the time of occurrence of this three different classes. The events in class 1 have occurred throughout the whole analyzed period. The events in class 2 and 3 occurred just over the period 1837–1985. Winter 1984/85 was the last winter when the ice cover duration was higher than 60 days. Figure 3b,c and d depicts the estimated occurrence rates of the ice cover duration for the three magnitude classes. For class 1 (Fig. 3b) there is a higher occurrence rate over the period 1837–1950, afterwards the occurrence rate stays at a constant level (class1 can occur once every 6.5–7 years). There is also a slightly decreasing trend in the occurrence of class 1 events, but the trend is not significant. For class 2 (Fig. 3c) and class 3 (Fig. 3d), the occurrence rates of ice cover duration exhibit similar features: high occurrence rates from 1837 until the beginning of 1900, followed by a sharp decreasing and significant downward trend after 1900 (99% significance level). Over the period 1837–1900, the highest occurrence rates are found for the class 2 events (~0.32/year), followed by class 3 events (~0.25 /year) and class 1 events (~0.24/year). The bootstrap confidence band (the pink shaded area in Fig. 3b,c and d) confirms the significance of these changes.

Over the analyzed period, both the winter mean temperature at Bucuresti and Sulina station as well as the ice cover duration data show significant trends and close years for the change in the mean: the winter mean temperature shows a positive and significant trend (99% significance level, Table S1), while the ice cover duration data shows a decreasing and significant trend (99% significance level, Table S1). One of the most striking features is the year of change in the mean identified for the mean winter temperature data and the ice cover data. By applying a Worsley likelihood test and a Cumulative deviation test^26^, we show that a significant jump, towards more positive temperatures, is found in 1947 at Bucuresti and Sulina stations. For the ice cover duration, both tests indicate a jump in the mean, towards less ice covered winters, at ~1943. The fact that the year of change in both winter mean temperature and ice cover duration are relatively close, could be an indication that the jump in the winter mean temperature was the driving factor of the sharp decrease in the ice covered data, especially in the second part of the 20^th^ century.

## Long-Term Reconstruction

For collecting information on ice covered winter, over our sector of interest, throughout the historical period, we relied mainly on old book entries and a collection of different sources (Table S2). Based on these documentary evidences we have reconstructed the occurrence of ice covered winters over the period 850 AD–1830 AD (Figure S2a). We have split our time series into the Medieval Warm period (MWP, ~850 AD–1250 AD) and the Little Ice Age (LIA, ~1251–1830). At the beginning of MWP there is an obvious decreasing trend in the occurrence rate of ice covered winters (Figure S2b). Between ~1200AD and 1600 AD there are small variations in the occurrence rate, the smallest occurrence rate being observed at ~1100AD and ~1200AD. The sharp increase in the occurrence rate at ~1650AD, significant at 90% level, could reflect the dry and cold climate of the Maunder Minimum period^27^. Between ~1750 AD and 1775 AD there is a small decrease in the occurrence rate of ice covered winters, followed again by a period with high occurrence rates from ~1775 AD until the end of the record. This increase in the occurrence rates took place during the Dalton Minimum period^28^, a period characterized by cold and dry winters. Similar to our results, Mudelsee *et al*.^29^, found also strong winter freezing over the Elbe and Oder rivers (Germany) during LIA. They also show that the freezing of these two rivers had an abrupt drop in the occurrence rate throughout the 20^th^ century, which they related mainly with regional warming. Some of the freezing events, in the lower part of the Danube River, during LIA, were so extreme that the freezing has extended from the Black Sea up to the Bosphorus (e.g. winters 1621, 1669, 1755, 1779, 1823)^30^.

## Ice Cover Duration – Temperature Relationship

**Figure 4:**
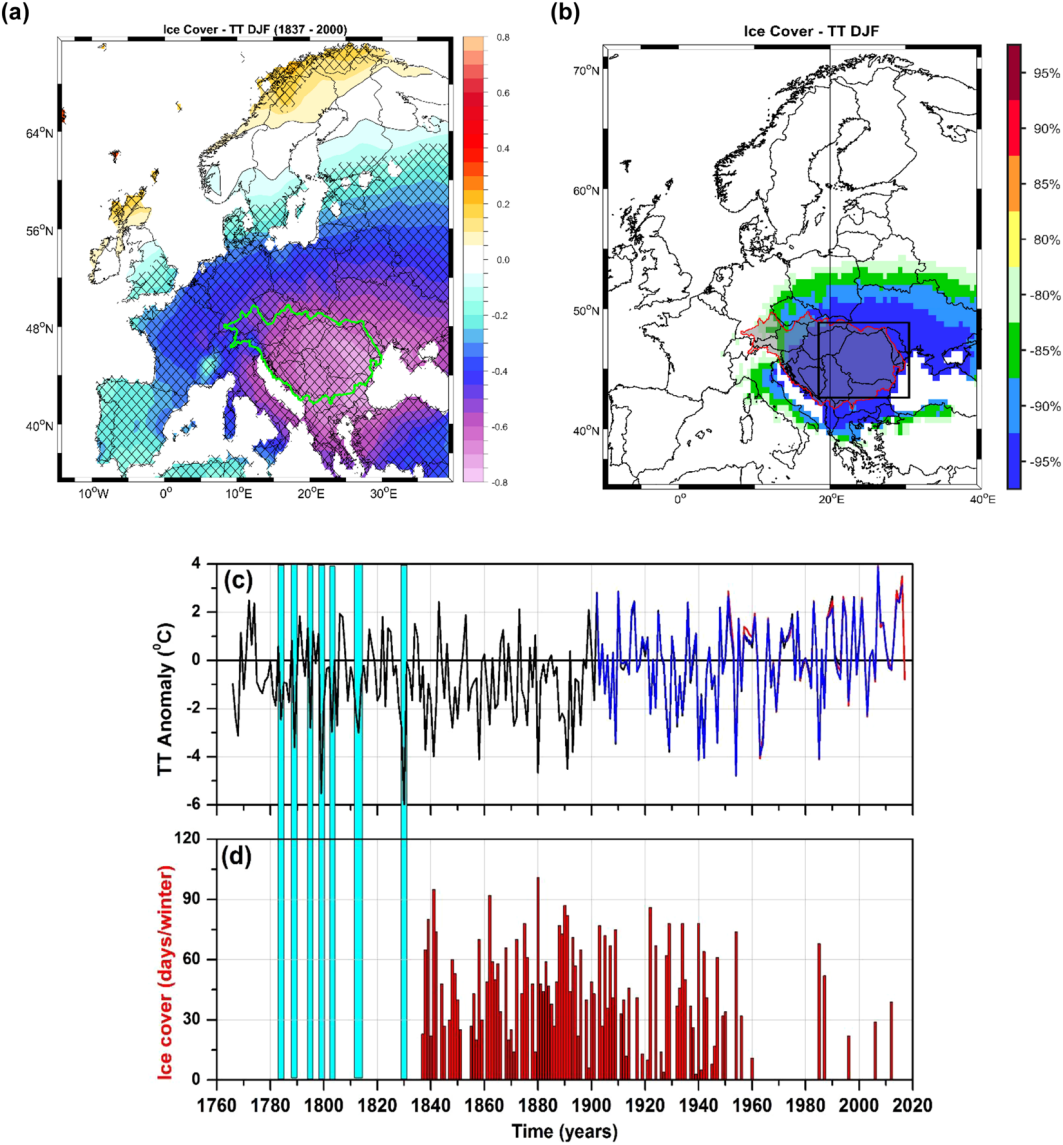
(**a**) The correlation map between the winter mean temperature (DJF) at European level^31^ and the number of days with ice cover over the period 1837–2000. (**b**) The stability maps (see Methods) between the winter mean temperature (DJF) at European level^31^ and the number of days with ice cover over the period 1837–2000; (**c**) Winter temperature anomaly averaged over the black square in (**b**) based on the Casty *et al*.^31^ reconstructed winter mean temperature (black line, 1766–2000), the CRUTS4.1 data^32^ (blue line, 1902–2016) and the E-OBS data^33^ (red line, 1951–2016) and (**d**) The number of ice cover days/winter at Tulcea station. The hatched areas in (**a**) indicate correlations significant at 95% significance level based on a *Student t-test*. The blue rectangles indicate extremely cold years and their correspondent in ice cover occurrence based on documentary data (Fig. S2).

The spatial relationship between the river ice duration and winter mean temperature was analyzed by employing a reconstructed temperature data set^31^ at European level. The relationship between the ice cover duration and winter mean temperature was analyzed by means of spatial correlation map (Fig. 4a) as well as stability maps (see Methods, Fig. 4b). The spatial correlation analysis revealed that the temperature signal recorded by the ice cover duration data has a wide spatial range. The area of significant and stable correlations extends over a large part of the central and eastern Europe. The highest correlations are found over the Balkan region. Based on the stability map (Fig. 4b) we have defined a temperature index averaged over the eastern part of Europe (black square in Fig. 4b), for different temperature data sets (Reconstruction^31^, CRU TS4^32^ and E-OBS^33^).

Figure 4c and d depict the temporal evolution of the winter mean temperature averaged over the eastern part of Europe (Fig. 4c) and the ice cover duration data (Fig. 4d). In general, extremely cold winters are always accompanied by ice covered winters. Over the period 1776–2000, the 10 coldest winters were recorded for the following years: 1798/1799, 1829/30, 1857/58, 1879/80, 1890/91, 1953/54, 1939/40, 1940/41, 1962/63 and1984/85. All these cold winters we accompanied by river ice cover >60 days/winter. The correlation coefficient between the winter mean temperature index and the ice cover duration data, over the period 1837–2000, is r = −0.73 (99% significance level).

Before 1837, we have indication just regarding the occurrence of ice cover in the lower part of the Danube River, but no indication regarding the duration. Nevertheless, the coldest winters (before 1837) identified in the winter mean temperature index have all a correspondent in the ice cover occurrence (blue bars in Fig. 4c and d). For example, for the winter 1829/30 (the coldest one on record) the documentary evidences show that “the winter was harsh, navigation on the Danube was interrupted by ice, there were heavy snows and the spring was delayed. The winter began in October and lasted until April”^34^. As such, due to its significant and stable relationship with the winter mean temperature, the ice cover duration record in the lower part of the Danube River has the potential to provide a powerful proxy temperature over the eastern part of Europe.

**Figure 5:**
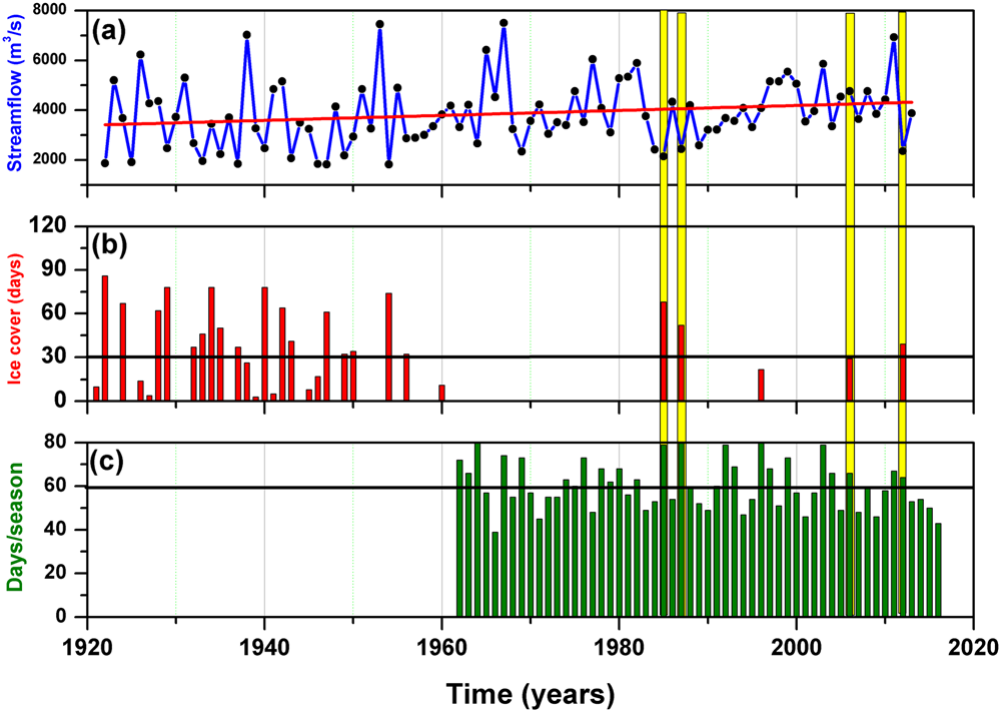
(**a**) The lowest discharge (minimum daily discharge computed from the daily streamflow data) throughout the winter months (December-January-February) measured at Ceatal gauging station over the period 1921–2013; (**b**) the number of ice covered days/winter at Tulcea station and (**c**) the number of frost days (the number of days when the daily minimum temperature was smaller than 0 °C) at Tulcea station over the period 1961–2016. The yellow bars indicate years with ice cover >30days/winter (middle panel) and their correspondent low flow values (upper panel) and the number of frost days/winter (lower panel).

Ice covered winters are also accompanied by low-flow situations in the lower part of the catchment area (Fig. 5a). The occurrence and magnitude of low flows during the ice season are a direct consequence of the ice cover and ice formation^35^. Over the period 1961–2016, for the winters when the ice cover duration was ≥30 days, there were always low-flow situation recorded (Fig. 5a) and the daily minimum temperature was below 0 °C for more than 60 days/winter (Fig. 5c). A slightly increasing trend (~99.4 m^3^/s/decade) is observed for the minimum winter discharge (the lowest daily discharge throughout the winter months (December-January-February)), in the second part of the 20^th^ century (Fig. 5a). This upward trend could be explained, at least partially, by the decreasing trend in the occurrence of ice cover over the same period of time. During the second part of the 20^th^ century, the number of days with daily minimum temperature <0 °C (frost days) shows a significant decreasing trend (Figure S3) at European level. Over our analyzed region, there is a reduction in the frost days of ~6 days/winter. This decreasing trend in the number of frost days/winter acts as an additional argument of the important role played by the warming trend in the winter temperature on the occurrence of the river ice cover, especially in the second part of the 20^th^ century.

## Large-Scale Drivers

**Figure 6:**
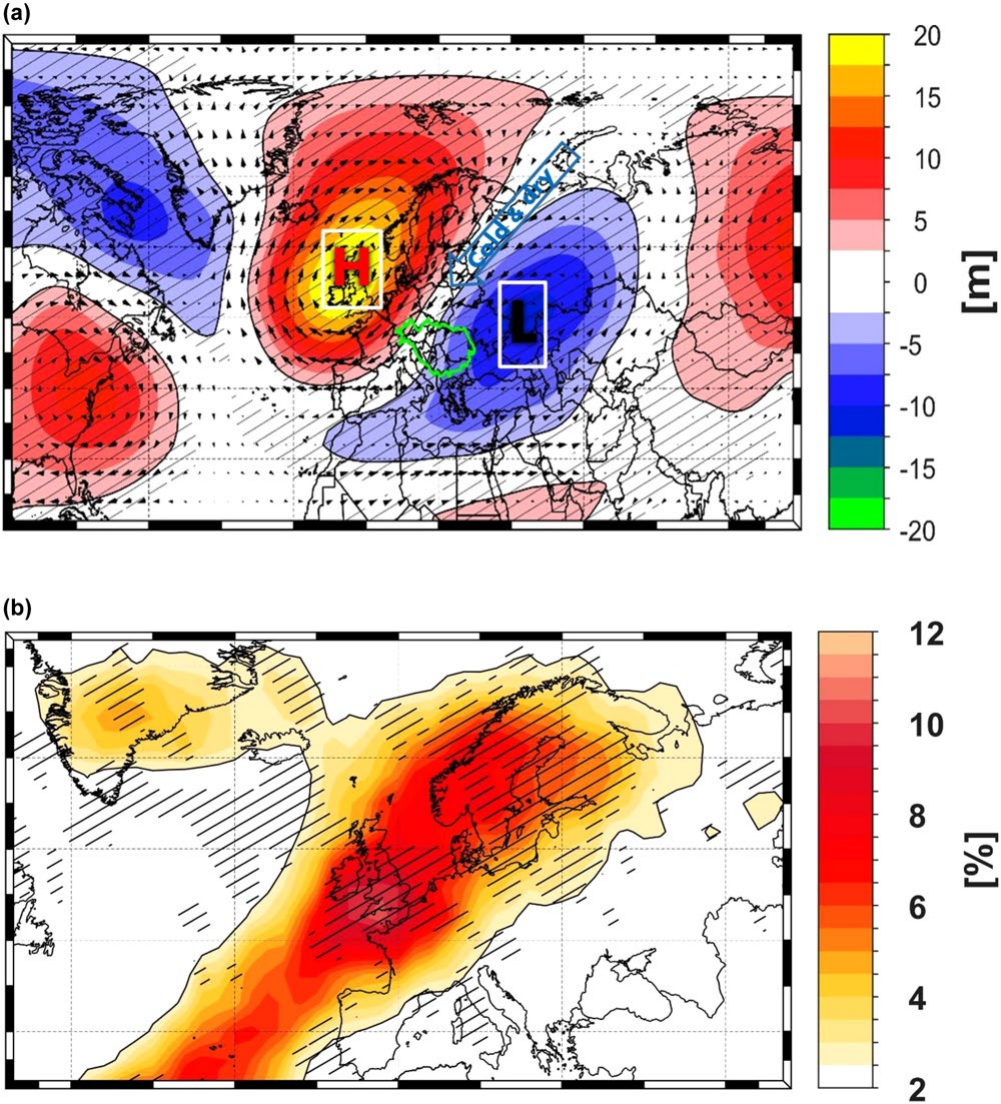
(**a**) The composite map of the winter (DJF) geopotential height and wind at 500 mb level^58^ for the years when the ice cover >30 days and (**b**) Winter (DJF) 2D atmospheric blocking frequency (see Methods) high composite maps for the years when the ice cover was >30 days. The hatching highlights significant anomalies at a confidence level of 95%.

A large part of the observed variability and trends in the global temperature is related to large-scale atmospheric circulation. The streamflow regime of Danube River, in the lower part, was found to be strongly influenced by the North Atlantic Oscillation/Arctic Oscillation teleconnection patterns^20,36^. In terms of the ice regime, the correlation over the period 1837–2016, between the winter NAO and ice cover data is very small and not significant (r = −0.2). In order to be able to infer the large-scale atmospheric drivers of the ice regime we have computed the composite map of the geopotential height and wind component at 500 mb, for the years when the ice cover duration was >30 days/winter (Fig. 6a). The resulting composite map shows both regional as well as large-scale features. Ice covered winters in the lower part of the Danube River, are associated with an anticyclonic circulation centered over the British Isles and a cyclonic circulation centered over the Black Sea and eastern part of Europe (Fig. 6a). These anomalous centers favor the advection of cold and dry air from the north-east towards the lower part of the Danube catchment area (Figure S4a). A similar patter in the large-scale atmospheric circulation was found to be associated with the occurrence of cold and dry winter over the whole Romanian territory^37^ (their Fig. 3b). Ice covered winters, tend also to occur in association with a significantly cold Black Sea and a warmer than normal north-east Atlantic Ocean (Figure S4b). Stanescu and Stanescu^22^ have also shown that, over the period 1929–1963, the ice covered winters in the lower part of the Danube Basin occurred under the influence of north, north-easterly or easterly flow, which brings extremely cold and dry air over the analyzed region. This can also be observed if we look at different extreme events, like the large-scale anomaly patterns and temperature anomalies for some of the coldest and ice covered winters: winter 1798/99 (Figure S5a and b), winter 1829/30 (Figure S5c and d), winter 1879/80 (Figure S5e and f) and winter 1928/29 (Figure S5g and h). For all these particular events, the flow is either north-easterly (winter 1798/99), easterly (winters 1829/30 and 1928/29) or northerly (winter 1879/80). All these events were also associated with extremely cold winters, at European level, with temperature anomalies up to −7 °C in some cases (winter 1798/99). The common feature of these events, is that in the case of the winter mean temperature anomalies, all of them show low temperatures over the regions were the correlation between ice cover duration and winter mean temperature shows the highest values (Fig. 4a,b).

Ice covered winters are also associated with a higher frequency of atmospheric blocking situations over the British Isles extending up to the Scandinavian Peninsula (Fig. 6b). Atmospheric blocking is a large-scale mid-latitude atmospheric phenomenon mostly associated with persistent quasi-stationary synoptic-scale high-pressure systems. Due to its persistent feature, it may cause large-scale circulation anomalies exerting a strong impact on weather patterns and is therefore often associated with significant climate anomalies, like cold spells, floods and droughts^38–40^. Rimbu *et al*.^39^ have shown that winters with increased atmospheric blocking over the British Isles and the Scandinavian Peninsula are associated with extremely cold winters over the central and eastern part of Europe (where the correlation between the ice cover duration and air temperature is the highest).

## Comparison with Other River Ice Cover Duration Data

Records of ice cover conditions are collected especially in the cold-regions countries (e.g. Canada, Russia, Norway), but for different purposes. Although river ice conditions have a significant importance for hydrology (i.e. ice jams are usually accompanied by extreme floods), large-scale analyses on this particular topic are almost non-existent^12^. Both the temporal and the spatial resolution are a problem for a hemispheric or continental evaluation of river ice conditions. The most comprehensive study, for the whole Northern Hemisphere, was published by Magnusson *et al*.^16^. In their study, Magnusson *et al*.^16^ showed that the freeze-up (break-up) dates have became 5.7 days/100 yrs later (6.3 days/100 yrs earlier). The rates of change were noted to correspond to an increase in the winter mean temperature by ~1.2 °C/100 yrs. Over the eastern part of Europe there are just a limited number of studies regarding the analysis of freshwater ice regime^14,15,41^. These studies are focused on the Danube River^15,41^, Drava River^15^ and lake Balaton^14^. For example, it has been shown that the freeze-up and break-up dates, for Danube River at Budapest station, have shifted later by 9–27 days/100 yrs and earlier by 7–13 days/100 yrs, respectively.

Figure S6a depicts the freeze-up date (blue), break-up date (red) and ice cover duration at Budapest station (Figure S6b) over the period 1775–201615 The correlation coefficient between the ice cover duration time-series at Tulcea and Budapest stations is r = 0.57 (99% significance level). In terms of duration, at Tulcea station the number of days with ice cover is, in general, much higher compared to Budapest station, mainly due to regional climate and geomorphological differences. The highest number of days with ice cover, at Budapest, was record during the winter 1879/80 (like in the case of Tulcea station – 101 days). The last year with ice cover at Budapest was during the winter 1984/85. Figure S7 depicts the occurrence rates for different ice cover duration classes (we used the same definition like in the case of Tulcea station). For class 1 (1 ≤ ice cover duration ≤60 days) the highest occurrence rate was found between 1830’ and 1930’s (~0.25 /yr), followed by a sharp decreasing trend over the last 80 years. For class 2 (60 < ice cover duration ≤90 days) and class 3 (ice cover duration > 90 days), the occurrence rates show an abrupt and significant downward trend starting ~1850’s. Although the two station are located on the main course of the Danube River, there are significant differences in the occurrence of the ice regime between the two stations. Ice cover duration and its occurrence rate tends to be higher at Tulcea station compared to Budapest station, throughout the whole analyzed region. This can be partially explained by different climatic drivers, different geomorphological feature as well as human induced changes (i.e. river regulations, hydropower use, water pollution). Nevertheless, the most important common feature is the abrupt decline in the occurrence of river ice cover, especially in the second part of the 20^th^ century. This abrupt drop in the ice cover occurrence, in the second part of 20^th^ century, has been observed also at Nagymaros, Mohacs and Komarom stations (all situated on the Danube River course) (Figure S8). The shift towards an almost ice free winters, in the lower part of the Danube River, has occurred almost at the same time with a sharp increase in the Northern Hemisphere winter mean temperature (Figure S8g). Between 1977 and 2016, the NH winter mean temperature anomalies are positive throughout all the years. Also the Eurasian lakes (i.e. Kallavesi, Oulujavri, Mjosa, Baikal, Table S3) show a significant decreasing trend in the ice cover duration, especially in the second part of the 20^th^ century (Figure S9, Table S3). The only exception is for Lake Balaton, where no significant trend was found for the ice cover duration.

## Conclusions

The pronounced warming of the NH land, in recent decades, is well documented^4^. Across many temperate areas the warming is typically greater in winter and spring^42,43^. Anthropogenic climate change is also expected to intensify the hydrological cycle^44^. As a consequence, hydrological flow regimes will be modified by climate change, through alterations in precipitation, temperature, snow and ice cover. Alongside with this changes, here we show that the ice cover data, from the lower part of Danube River, revels a significant decreasing trend, after 1950’s, in the occurrence rate of ice covered winters. Over the last ~60 years, there were just ten winters when river ice cover occurred. Before 1950’s ice cover was occurring almost every winter, with small exceptions. This significant decreasing trend in the number of ice covered winters follows the general NH pattern. River across the NH have experienced similar trends regarding the length of the ice cover duration^14,15,45–47^. In terms of freeze-up and break-up dates, our time series does not follow the general NH pattern. At NH scale, there is an overall trend towards later ice freeze (~0.57 days/decade) and earlier ice breakup (~0.63 days/decade)^6^. For our region, no significant trend/change was found in the freeze-up and break-up dates (Fig. 2a).

The decrease in the occurrence of ice covered winters over the last ~60 years, could be explained, at least partially, by the increase in the winter mean temperature (both at European and local scale) and the significant decrease in the occurrence rate of cold winters (Figure S4). We have also tested the influence of other potential driving factors (i.e. the NAO, solar forcing, Cernavoda nuclear power plant, among others), but no significant relationship was found between the ice cover duration and the aforementioned drivers (not shown). For example, the Cernavoda power plant, which is situated upstream of Tulcea, became functional at the beginning of the 1980’s, which is ~30years later after the change in the mean occurrence rate of ice covered winters. Although NAO is considered one of the most important drivers of winter European climate^48^, no significant relationship between the winter NAO index and ice cover duration time series has been identified. Ice covered winter in the near vicinity of the Black Sea and Danube Delta are occurring in association with a negative SST anomalies over the Mediterranean Sea and Black Sea and with an Rossby wave-like structure in the large-scale atmospheric circulation, characterized by enhanced blocking activity over the British Isles and Scandinavian Peninsula and a low pressure center over the eastern part of Europe. This pattern favors the advection of cold and dry air from the north or north-east, which leads to the occurrence of river ice covered winters in the lower part of the Danube River.

To have a clear picture regarding the drivers of the significant decrease in the occurrence of ice covered winters, after the beginning of 1950’s, we have first tested if there were significant changes in the atmospheric circulation. By performing a trend analysis and cluster analysis (not shown), we found that no significant changes have occurred in the large-scale atmospheric circulation. The dipole-like structure (Fig. 6a) which favors the occurrence of ice covered winter at Tulcea, was present also after the 1950’s and no trend or change has been observed in the occurrence rate of this particular pattern. Further, we have tested if there were significant changes in the winter mean temperature, not just locally, but also at a larger spatial scale. As such, we have computed the difference map, in the winter mean temperature, for one period characterized by a high occurrence rate of ice covered winters (1901–1950) and one period characterized by a reduced occurrence rate of ice covered winters (1971–2016) (Figure S10a). For the second period (1971–2016) there is a hemispheric increase in the winter mean temperature, with the strongest increase over the Eurasia, Canada and Greenland. Also over the eastern part of Europe there is a difference of ~+1.5° between the two periods. Overimposed onto these significant changes in the winter mean temperature, starting 1980’s, there is also a significant positive trend in winter mean SST in the Black Sea (Figure S10b) and in the vertical integral of water vapor over the Atlantic Ocean basin, the Arctic basin, the Scandinavian Peninsula and western part of Russia (Figure S10c). The increasing winter mean temperature over the eastern part of Europe and western Russia corroborated with a tendency towards wetter winters, could explain, at least partially, the downward trend in the occurrence of ice covered winter at Tulcea. Even a minor increase in the mean air winter temperature can cause significant changes in rivers and lakes freeze-up and break-up dates, as well as in the ice cover duration^49,50^.

An additional factor contributing to the significant decrease in the occurrence of river ice after the beginning of 1950’s could be related to water pollution (e.g. waste water and thermal pollution). Anthropogenic influences on the occurrence of ice regime, for different water bodies, have been already observed for the Lower Vistula^51^, the Silesian Upland (southern Poland)^52^ and the Danube River at Budapest station^41^. Between 1837–1950, a mean winter temperature of −0.54 °C was needed to induce ice cover occurrence at Tulcea station, while a mean winter temperature of −1.05 °C was needed after the beginning of the 1950’s (Figure S11). This suggests that for the occurrence of river ice much colder temperatures were needed over the last six decades when compared with the period 1837–1950. Thus, anthropogenic effects should also be considered in future studies regarding the occurrence of river ice.

In a long-term context, here we show that the ice cover occurrence rate follows the variability of winter mean temperature throughout the MWP and LIA. Warmer periods, like the MWP, are characterized by a reduced ice cover occurrence rate, while colder periods, like LIA, are characterized by higher ice cover occurrence rate. As such, historical and observational data regarding the occurrence rate of ice cover could provide us with valuable information on past and present changes in the large-scale climate. The high correlation between the number of ice cover days and the winter temperature over the central and eastern part of Europe, reveals that the ice regime can be used as a proxy for the winter temperature over this region.

Coupled atmosphere-ocean general circulation models predict that rising greenhouse gases will lead not only to increases in the mean air temperature, but also a warming of the northern hemisphere lakes and rivers^53^. The projected median changes of the FD simulated in the CMIP5 ensemble runs^54^, for the RCP8.5 scenario (worst-case scenario), are shown in Figure S12. A significant decrease in FD (number of days when T_n_ < 0 °C) is obvious at hemispheric scale. The decrease in the number of FD is particularly strong over the western part of North America and the central and eastern part of Europe. In the RCP8.5 scenario the reduction of FD, over the period 2051–2100 relative to the period 1961–1990, is up to ~60days/year in the central and eastern part of Europe. Overimposed on these changes, an increase in the winter mean air temperature in the range of ~2.5 to 5.5 °C (RCP8.5) is projected until the end of the 21^st^ century, over the eastern part of Europe^55^ together with an increase in the surface water temperature of ~1 °C in the lower part of the Danube River^56^. Under these conditions, one expects that by the end of the 21^st^ century the ice regime in the lower part of the Danube River will be drastically reduced, due to the fact that, over the analyzed region, river ice cover appears just when the winter mean temperature drops bellow 0 °C. Moreover, winter with ice cover duration >60 days/winter occur just for winter mean temperatures < −2 °C (Figure S13).

A decrease in the occurrence of icy winters corroborated with an increase in the mean air temperature and the water surface temperature of lakes and rivers will hinder the formation of ice cover, thus leading to important consequences for the water quality and management, biodiversity, ecology, inland waterway transport and tourism, among other sectors^57^. Thus, advancing the understanding of climate-induced changes to lake and river ice and the subsequent effects will require improvements for the monitoring, predictive modeling, and assessments of adaption options. Such an approach will require additional data collection, further research focused on river ice processes, more detailed regional river ice trend analyses and additional studies related to potential climatic changes that can be expected to occur in regions with lake and river ice cover.

## Data and Methods

### Data

For the Northern Hemisphere large-scale atmospheric circulation, we use the daily and monthly means of geopotential height at 500 mb level, as well as the zonal and meridional wind at the same level, from the twentieth century reanalysis project on a 2° × 2° grid^58^. Global sea surface temperature (SST) is extracted from the Hadley Center Sea Surface Temperature data (HadSST)^59^. This dataset covers the period 1871–2016 and has a spatial resolution of 2° × 2°.

### Occurrence rate estimation

The occurrence rate of the time-dependent extreme events can be computed as^60^:

1 $$\lambda ({\rm{t}})={h}^{-1}\sum _{n=1}^{m}K(\frac{t-{T}_{n}}{h})$$

where *T*_*n*_ is the timing of the n^th^ ice event with unit of year; m is the number of ice events occurrence; K() is the Gaussian kernel function and h is the width of the kernel function (h = 30 years). Confidence intervals (90%) around λ(t) were determined using a bootstrapped technique: N simulated ice events were drawn from *T*_*n*_ with replacement and simulated *λ* calculated. This procedure was repeated 5000 times and a percentile-t confidence band was calculated. The trends in the occurrence rate were confirmed using the Cox and Lewis statistical test^61^.

### Trends and change points

The rank-based non-parametric Mann-Kendall (M-K) test^62,63^ and Spearman’s Rho, which are less sensitive to outliers than parametric statistics, were used. To avoid the influence of serial persistence on M-K test results, the modified M-K (MMK) trend test was used, using the computation algorithm discussed by Hamed and Rao^64^.

### Stability Maps

The stability map analysis is based on a methodology similar to the one used for the monthly and seasonal prediction of Elbe river streamflow^65–68^. The basic idea of this procedure is to identify regions with stable teleconnections between two variables. The ice cover data has been correlated with the winter mean air temperature, in a moving window of 31 years. The results remain qualitatively the same if the length of the moving window varies between 15 to 35 years. The statistical significance of the correlation coefficient is tested using a *Student* t-test. The correlation is considered to be stable for those grid-points where the ice cover data and winter mean temperature are significantly correlated at 95%, 90%, 85% and 80% level for more than 80% of the 31-year windows, covering the period 1837–2000. The regions where correlation is positive and stable will be represented as dark red (95%), red (90%), orange (85%) and yellow (80%) on a global map. The regions where correlation is negative and stable will be represented as dark blue (95%), blue (90%), green (85%) and light green (80%) (see Fig. 4b). Such maps will be referred in our study as stability maps and their structures remain qualitatively unchanged if the significance levels that define the stability of the correlation vary within reasonable limits.

### Computation of the 2D blocking frequency

As a measure of local blocking frequency we have used the two-dimensional (2D) blocking index^69^. To compute the 2D atmospheric blocking index we used the winter daily 500 mb geopotential height (Z500). This data set was extracted from the NCEP/NCAR reanalysis data^58^ for the 1851–2014 period. The 2D blocking index is an extension of the one-dimensional blocking index^70^ to a two-dimensional map of blocking frequencies at every grid point. For each grid-point the southern gradient (GHGS) and the northern gradient (GHGN) are evaluated as follows:

2 $$GHGS=(Z({\phi }_{0})-Z({\phi }_{0}-15^\circ ))/15^\circ $$

3 $$GHGN=(Z({\phi }_{0}+15^\circ )-Z({\phi }_{0}))/15^\circ $$

where *φ*_0_ is the latitude of the considered grid point varying from 35°N to 75°. For each winter we calculate the ratio between the number of days when a certain grid point was blocked, i.e. the conditions GHGS > 0 and GHGN < (−10m/°.lat) are simultaneously satisfied for at least five consecutive days, and the total number of winter days (90 days). Because we have used Z500 data for 20°N–90°N, the blocking field extends from 35°N to 75°N.

### Composite analysis

To identify the physical mechanism responsible for the connection between the ice cover duration and the large-scale atmospheric circulation (Fig. 6a), the 2-D atmospheric blocking frequency (Fig. 6b), the vertically integrated water vapor transport (WVT – Figure S4a) and the winter sea surface temperature (SST, Figure S4b) we constructed the composite maps between the time series ice cover duration for the years when the values of the ice cover duration were higher than 30 days/winter. This threshold was chosen as a compromise between the strength of the climate anomalies associated to ice cover duration and the number of maps which satisfy this criterion. Further analysis has shown that the results are not sensitive to the exact threshold value used for our composite analysis (not shown). We have computed composite maps, instead of correlation maps, because the former considers the nonlinearities included in the analyzed data. The significance of the composite maps is based on a standard t-test (confidence level 95%).

## Electronic supplementary material

Supplementary file
