# Supplementary material for: Vanishing river ice cover in the lower part of the Danube basin – signs of a changing climate
Source: Sci Rep. 2018 May 21;8:7948. doi: 10.1038/s41598-018-26357-w (PMC5962587; doi:10.1038/s41598-018-26357-w)
Supplement: Supplementary file 1 — Supplementary file [file 41598_2018_26357_MOESM1_ESM.pdf]

Supplementary file

Vanishing river ice cover in the lower part of the Danube basin – signs of a changing climate

M. Ionita[1], C.-A. Badaluta[1,2,3], P. Scholz[1] and S. Chelcea[4]

[1]Alfred Wegener Institute, Helmholtz Center for Polar and Marine Research, Bremerhaven, Germany
[2]Stable Isotope Laboratory, Ștefan cel Mare University, Suceava, Romania
[3]Department of Geography, Stefan cel Mare University, Suceava, Romania
[4]National Institute of Hydrology and Water Management, Bucharest, Romania

Correspondence to: Monica Ionita (Monica.ionita@awi.de)

Address:
Alfred Wegener Institute Helmholtz Centre for Polar and Marine Research
Bussestrasse 24
D-27570 Bremerhaven
Telephone: +49(471)4831-1845
Fax: +49(471)4831-1271

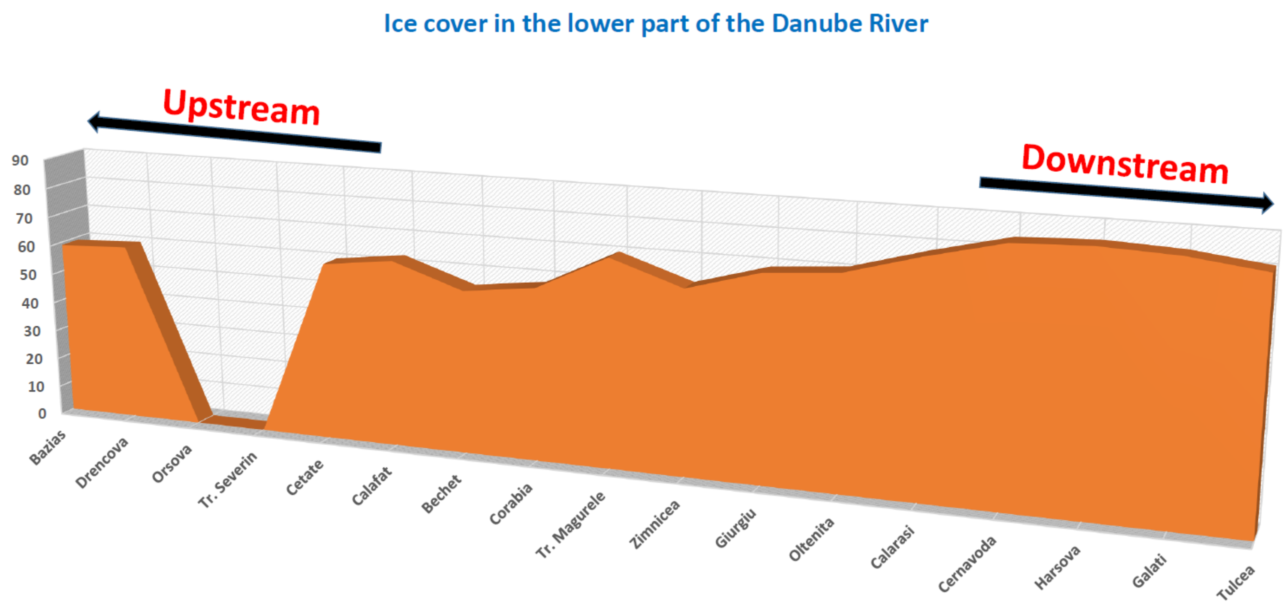

Figure S1. The ice cover regime over the Romanian part of the Danube river. The number of ice covered days increases downstream. The station with the highest number of river ice are found in the vicinity of the Danube Delta (Galati and Tulcea stations).

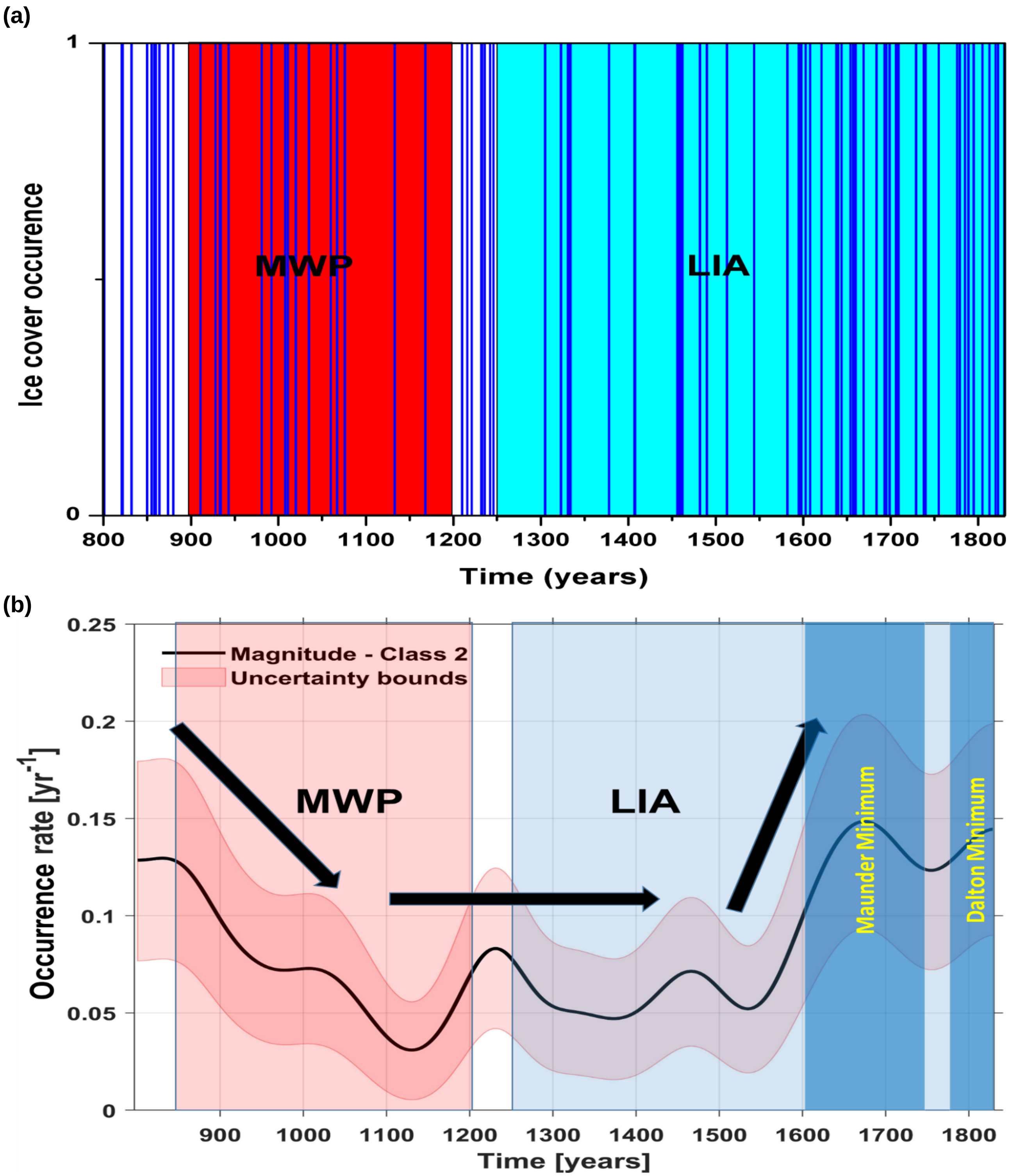

Figure S2. (a) Long-term reconstruction of the ice cover winters in the lower part of the Danube river based on documentary evidences, over the period 850 – 1830 and (b) the occurrence of ice cover over the period 850 – 1830. The black lines in (b) indicate the occurrence rate and the magenta shaded areas indicate the 90% confidence bands. The black arrow indicates the sign of the trend. In (a) and (b) MWP = Medieval Warm Period and LIA = Little Ice Age.

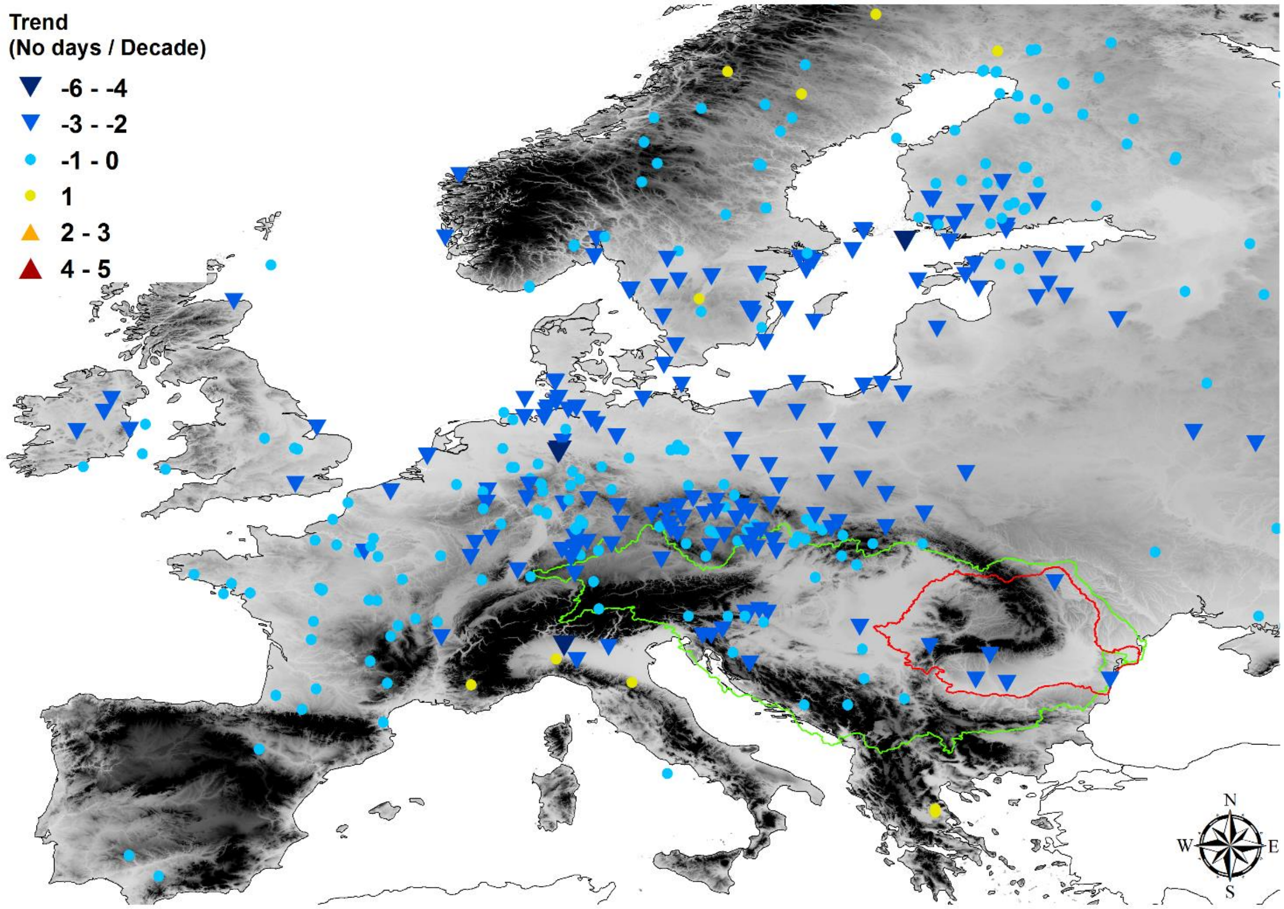

Figure S3. Winter (DJF) frost days[1] trend over the period 1951 – 2017. Downward triangles indicate significant (95% confidence level) and negative trends, upward tringles indicate significant (95% confidence level) and positive trends, blue circles indicate negative and non-significant trends and yellow circle indicate positive and non-significant trends.

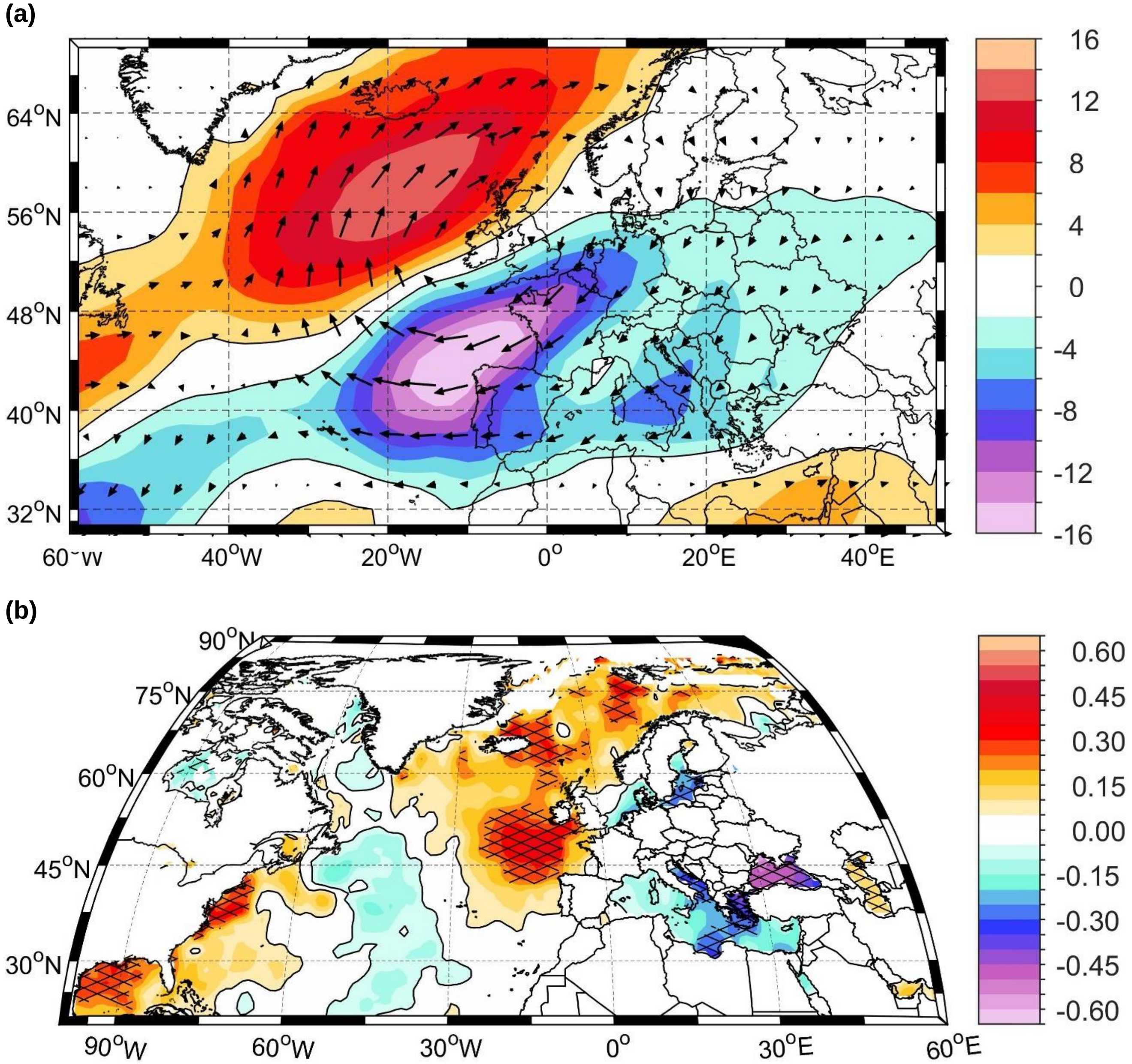

Figure S4. (a) The composite map of the winter (DJF) vertically integrated water vapor transport (WVT)[2] for the years when the ice cover duration > 30 days and (b) as in a) but or the winter SST[3]. The hatching highlights significant anomalies at a confidence level of 95%. Units: a) 10^{-6} kg m s^{-1} and b) K.

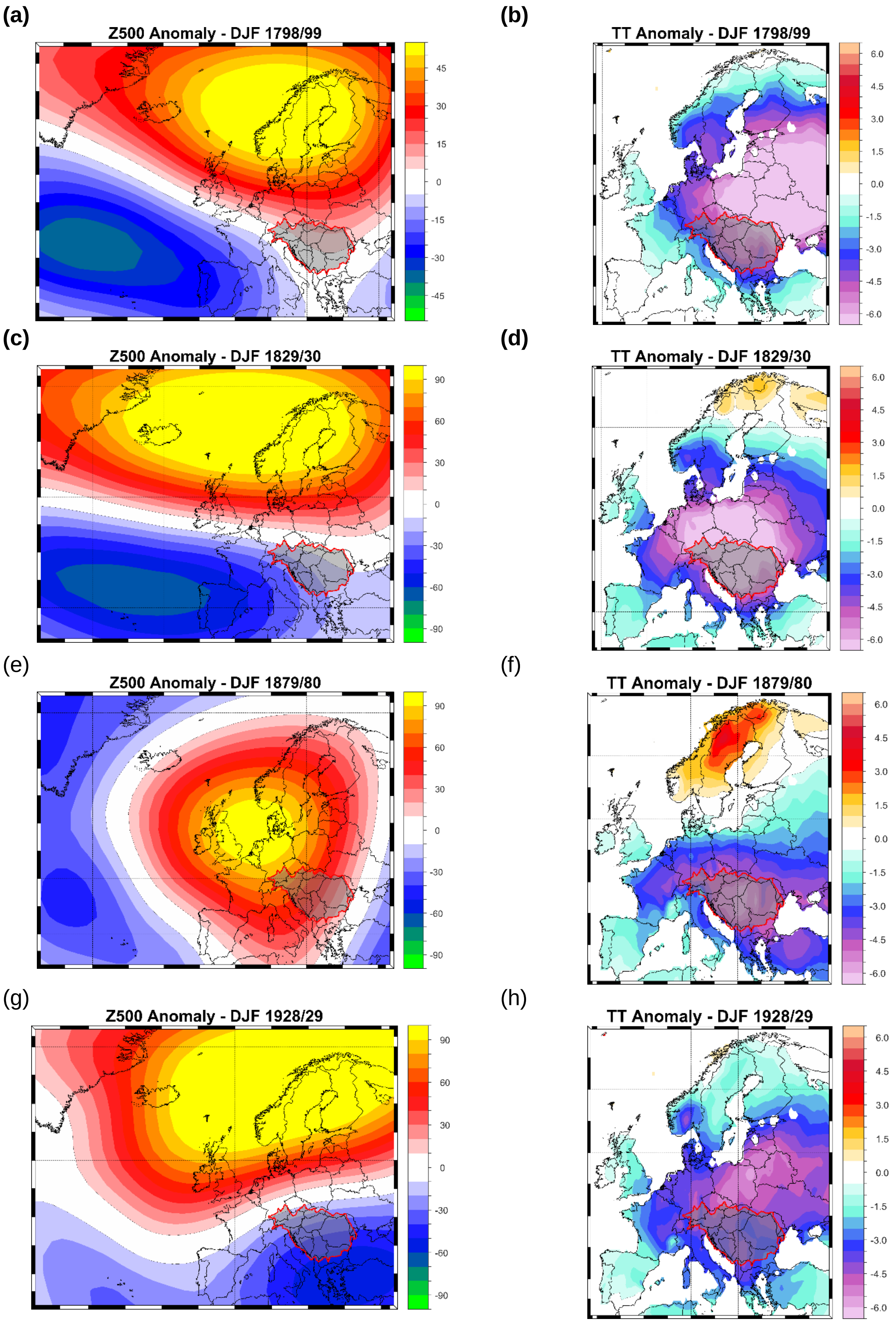

Figure S5. Z500 anomalies[5] (left panels) and TT[6] anomalies (right panesl) for selected extreme cold and icy winters: (a) and (b) winter 1798/99; (c) and (d) winter 1829/30; (e) and (f) winter 1879/1880 and (g) and (h) winter 1928/29. Units: Z500 (hPa) and TT (^{0}C).

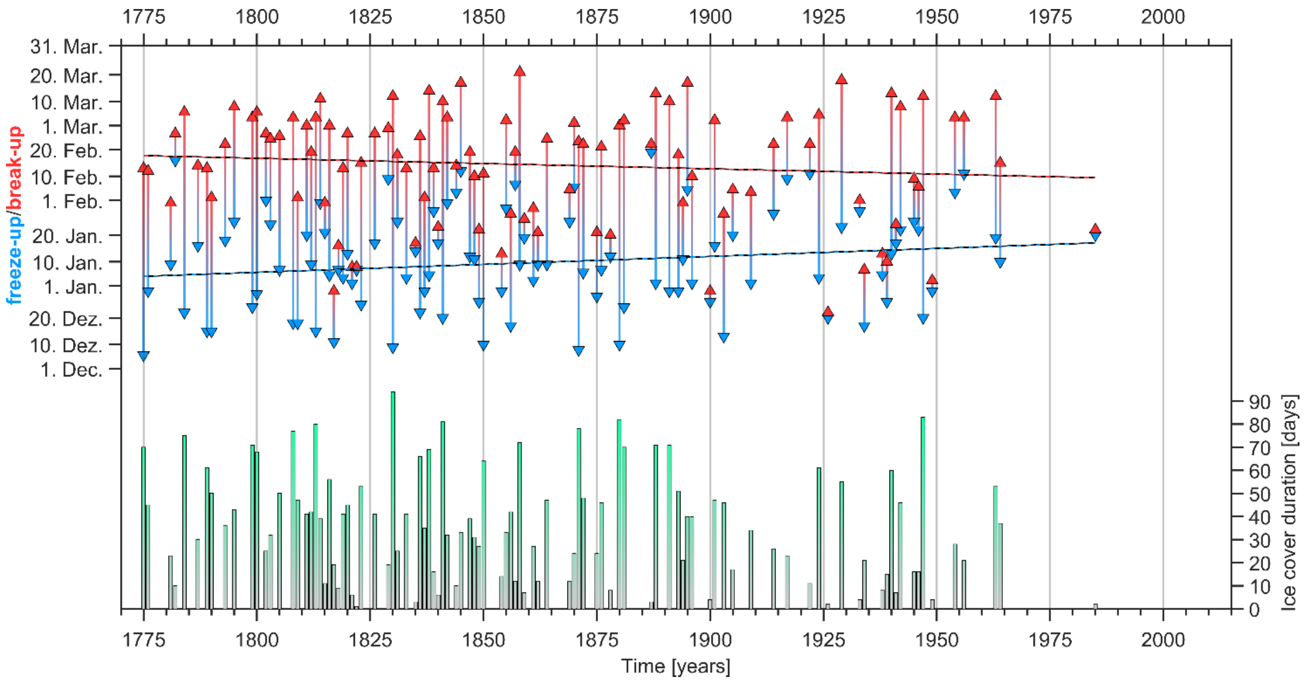

Figure S6. (a) The date of freeze-up and break-up and (b) the ice cover duration (days/winter) at Budapest station[6]. Blue arrows indicate the freeze-up dates and red arrows indicate the break-up dates.

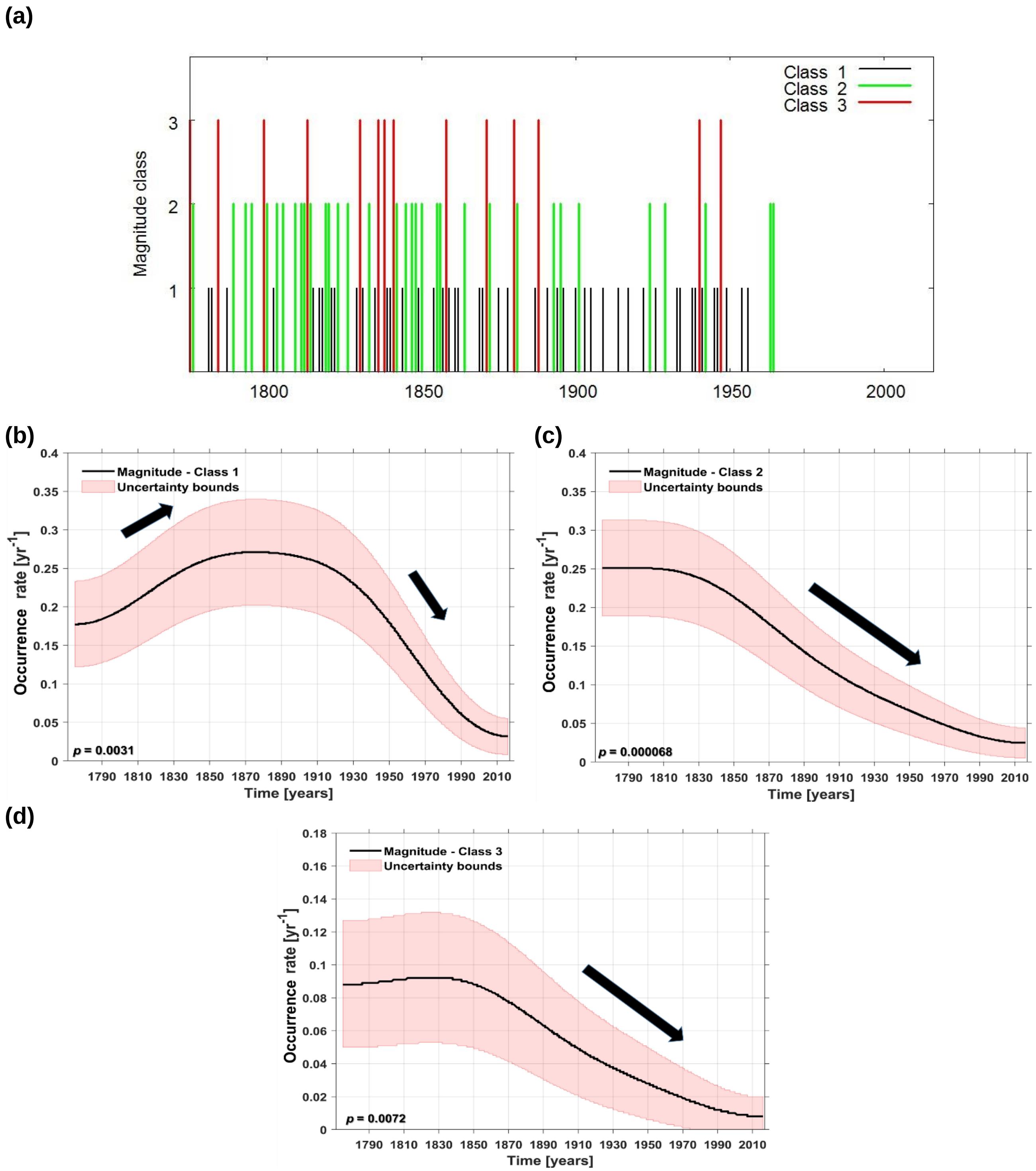

Figure S7. (a) Ice cover magnitude at Budapest station: black bars (class 1) indicate the years with ice cover between 1 and 60 days/winter, green bars (class 2) indicate the years with ice cover between 61 and 90 days/winter and red bars (class 3) indicate the years with ice cover > 90 days/winter; (b) Occurrence of ice cover from class 1; (c) occurrence of ice cover from class 2; (d) occurrence of ice cover from class 3. (a) was analyzed using a Gaussian kernel, a bandwidth of 35 years and bootstrap simulations (see Methods). The black lines in (b), (c) and (d) indicate the occurrence rate and the magenta shaded areas indicate the 90% confidence bands. The black arrows indicate the sign of the trend (downward for all classes). The trend is significant for all classes.

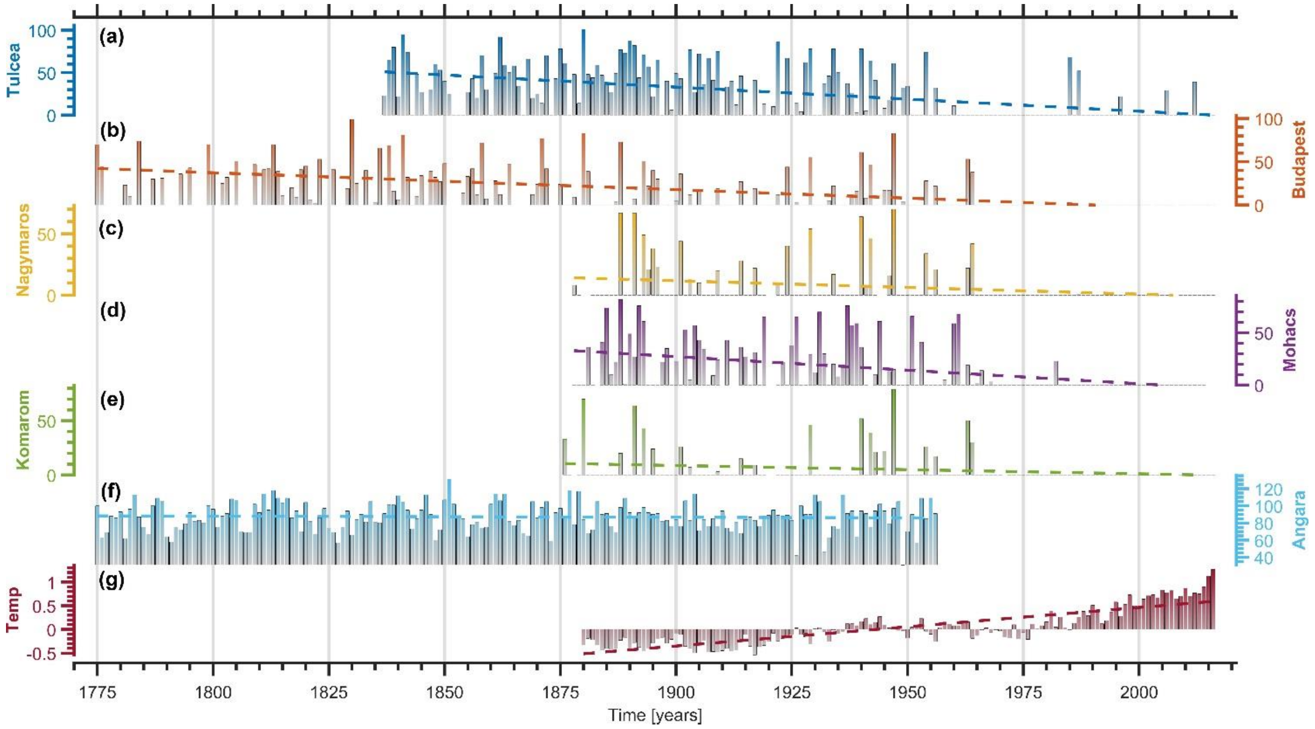

Figure S8. River ice cover duration at a) Tulcea (Danube River); (b) Budapest (Danube River); (c) Nagymaros (Danube River); (d) Mohacs (Danube River); (e) Komarom (Danube River); (f) Angara River and (g) Northern Hemisphere annual mean temperature anomaly. In (g) the annual mean temperature anomaly is computed relative to the reference period 1971 – 2000. Units: river ice cover (days) and NH temperature anomaly (^{0}C).

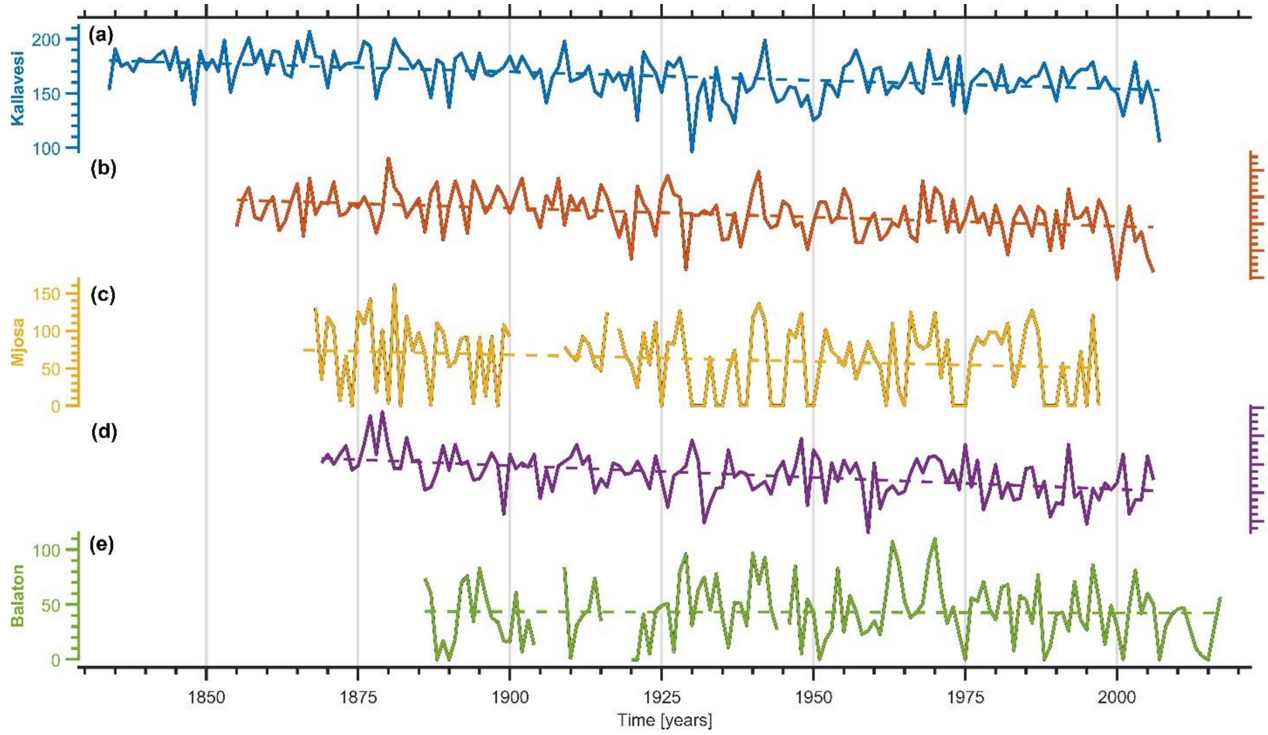

Figure S9. Lake ice cover duration at: (a) Lake Kallavesi (Finland); (b) Lake Oulujavri (Finland); (c) Lake Mjosa (Norway); (d) Lake Baikal (Russia) and (e) Lake Balaton (Hungary). Units: Lake cover (days).

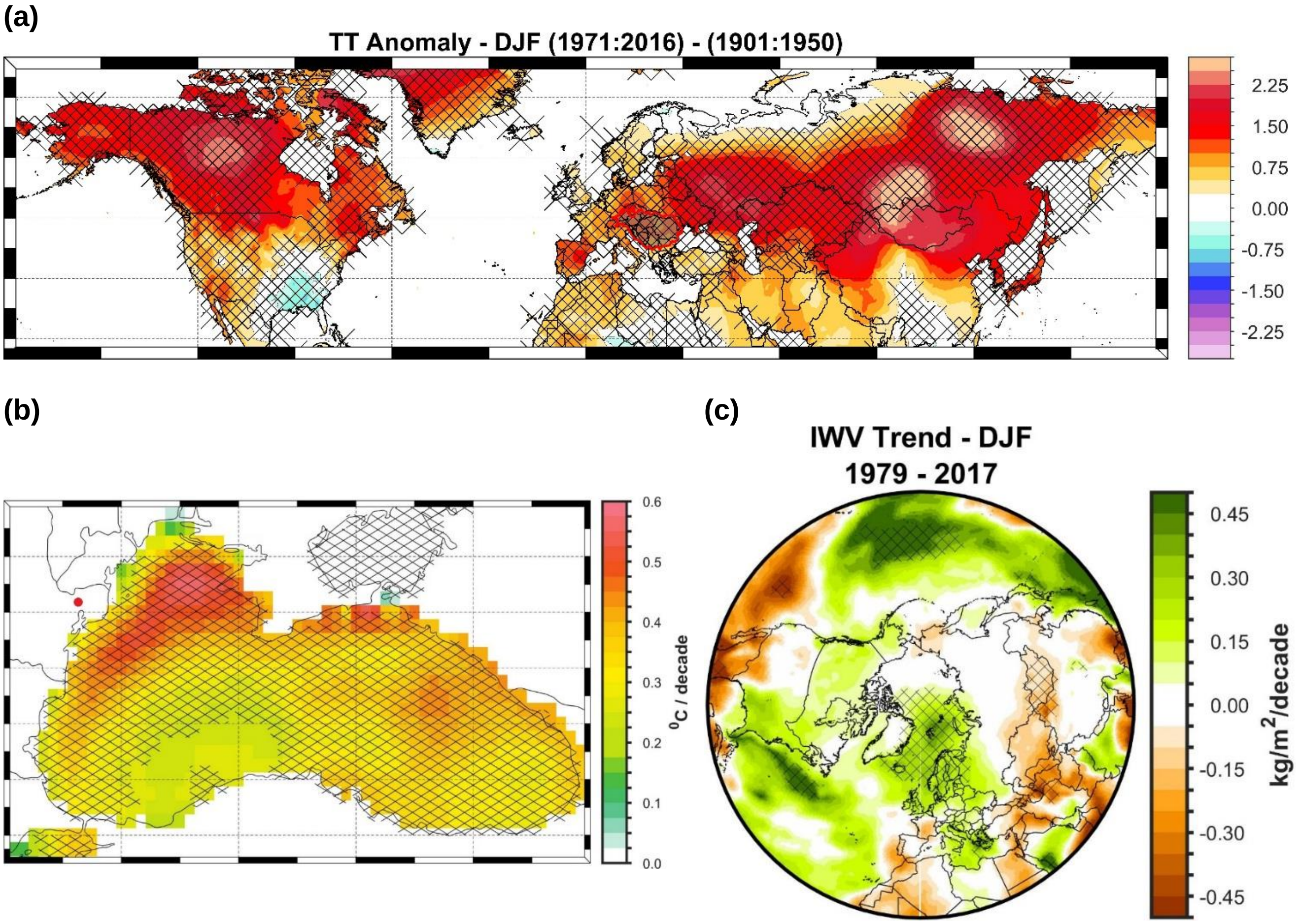

Figure S10. (a) Winter mean temperature anomalies (^{0}C) [(1971 – 2016) relative to the period (1901 – 1950)][7]; (b) Winter SST trends in the Black Sea over the period 1981 – 2017[8] and (c) Winter total integrated water vapor transport trend over the period 1979 – 2017[9]. The hatched areas in (a) indicate anomalies significant at 95% significance level based on a *Student t-test*. The hatched areas in (b) and (c) indicate significant trends at 95% significance level based on a *Student t-test*.

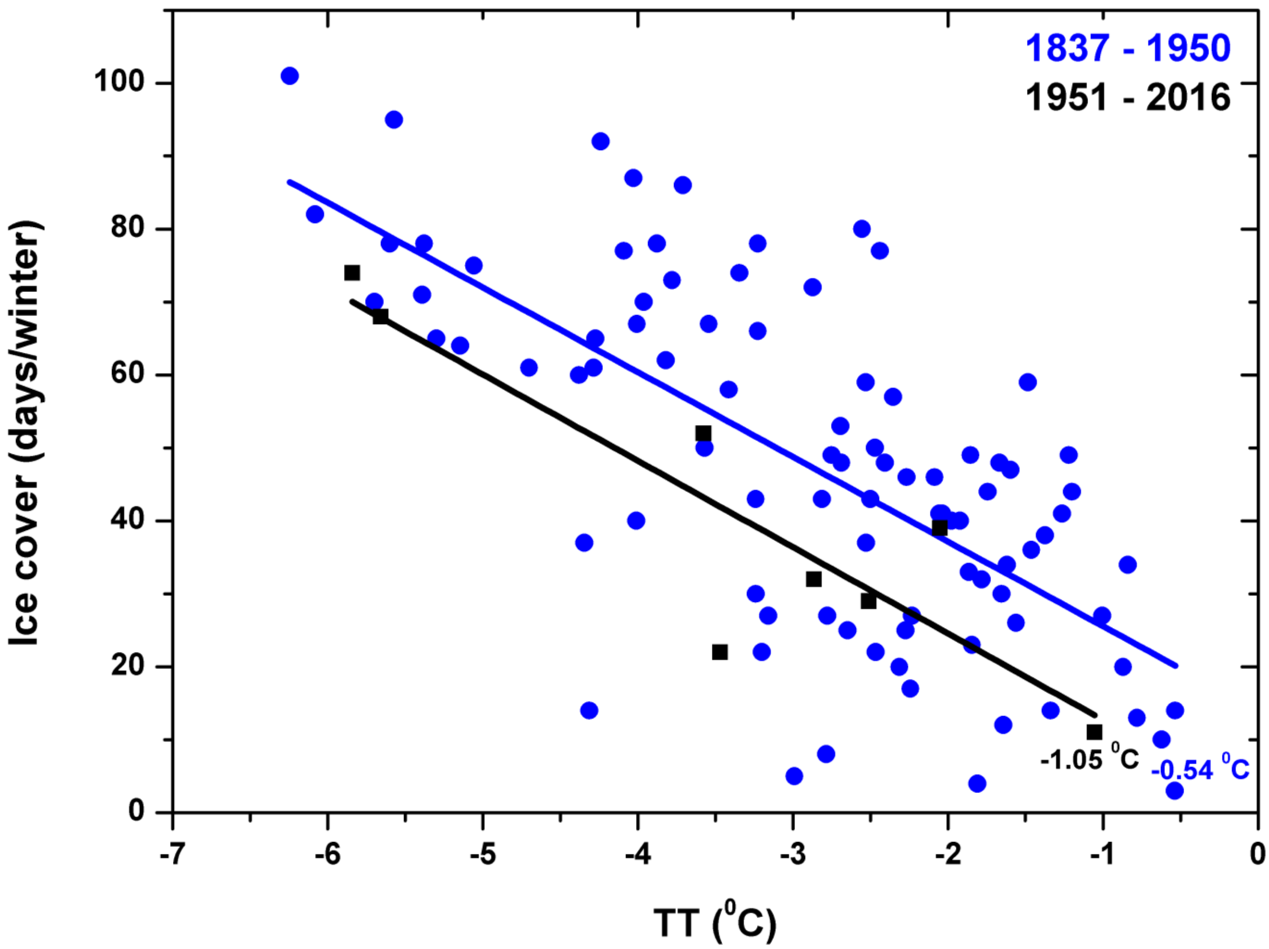

Figure S11. Relationship between the ice cover duration at Tulcea station and mean winter temperature calculated over two periods: 1837 – 1950 (blue) and 1951 – 2016 (black).

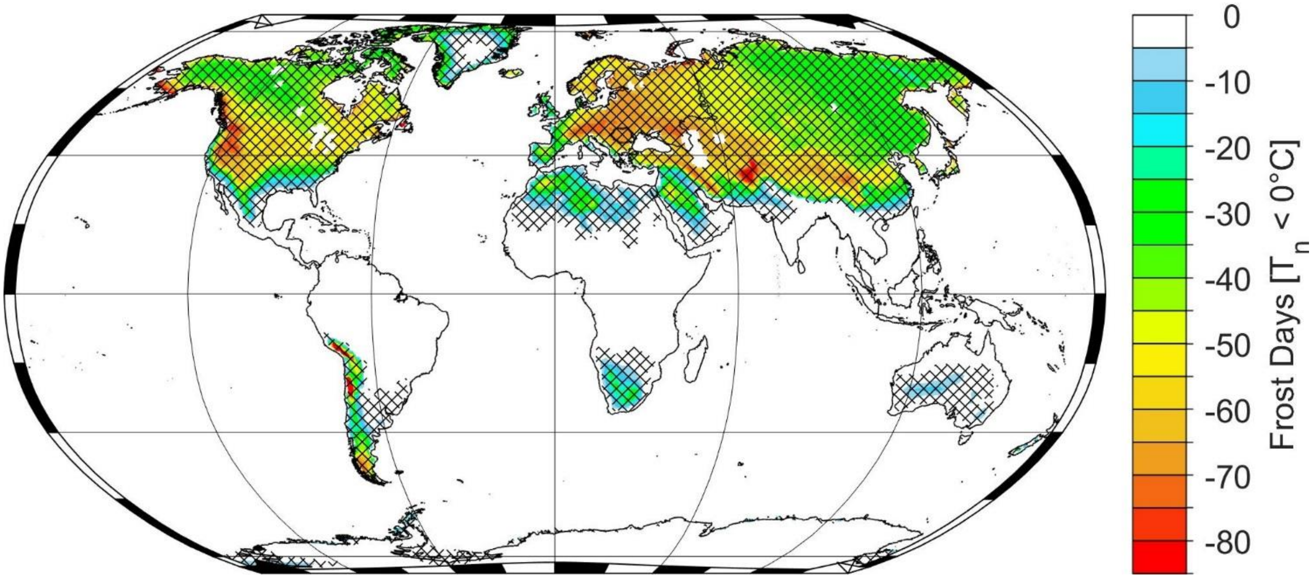

Figure S12. The multimodel median of temporally averaged changes in the Frost Days[11] over the time period 2051 – 2100 displayed at the difference (in number of days/year) relative to the reference period 1961 -1990 for RCP8.5. Stippling indicates grid points with changes that are significant at the 95% significance level.

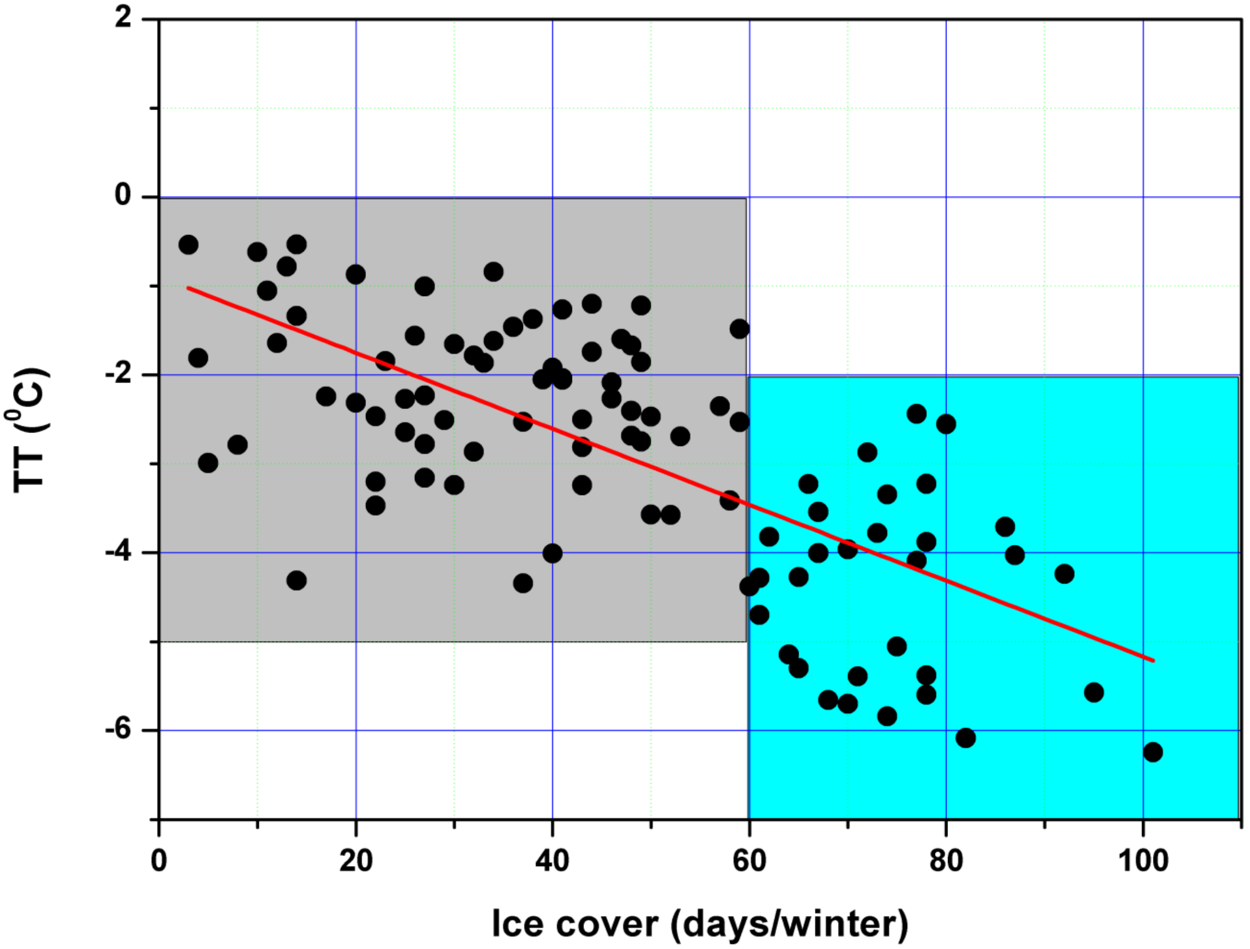

Figure S13. The scatter plot of the ice cover duration at Tulcea station (X – axis) as a function of the winter mean temperature (Y – axis). The red line indicates the linear regression line. Days with ice cover duration >60 days occur just at TT < -2^{0}C.

Table S1. Statistical test for the trend and jump in the mean for the temperature and ice cover data.

Test	**Bucuresti**			**Calarasi**			**Sulina**			**Ice cover**		
	**Obs.**	**Year of change**	**Signif.**	**Obs.**	**Year of change**	**Signif.**	**Obs.**	**Year of change**	**Signif.**	**Obs.**	**Year of change**	**Signif.**
**Mann-Kendall**	**3.004**		***	**1.097**		NS	**2.693**		***	**-7.185**		***
**Spearman's Rho**	**3.043**		***	**1.263**		NS	**2.688**		***	**-4.798**		***
**Linear regression**	**3.029**		***	**1.162**		NS	**2.63**		***	**-7.936**		***
**Cumulative deviation**	**1.750**	**1947**	***	**0.965**	**1947**	NS	**1.464**	**1947**	**	**3.56**	**1943**	***
**Worsley likelihood**	**3.671**	**1947**	***	**1.967**	**1947**	NS	**3.004**	**1947**	**	**8.57**	**1943**	***

*** - 99% significance level, ** - 95% significance level, * - 90% significance level, NS – not significant

Table S2. List with the source of the documentary evidences used for the long-term reconstruction of river ice occurrence rates over the period 800 – 1830.

Source	Year	Title of publication	Type	Journal/Edited	Volume	Pages
Mihăilescu, C.	2004	Clima și hazardurile Moldovei – evoluția, starea, predicția	Book	Publised by Licorn		191
Topor, N.	1964	Ani ploioși și secetoși	Book	Institutul Meteorologic Bucuresti		304
Teodoreanu, E.	2012	Apercu sur le climat des siecles passes sur le territoire de la Roumanie	Article	RRG.	56 (1)	71-86
Teodoreanu, E.	2013	Hydro-climatic events during the Little Climatic Optimum in Romania	Article	RRG.	57 (1)	3 - 8
Teodoreanu, E.	2014	The lower Danube in pages of history and literature	Proceeding	Inter. Conf. Water resources and wetland		577-583
Teodoreanu, E.	2014	Little Ice Age in Romania in the vision of a syrian traveler	Article	PESD	8 (1)	139-145
Teodoreanu, E.	2016	Freezing lower Danube during the last two millennia	Proceeding	Inter. Conf. Water resources and wetland		128-132
Teodoreanu, E.	2017	Little climate optimum in the Carpathian-Danubian-Pontic space	Article	PESD	11 (1)	91-98
Yavuz, V. et al.	2010	The frozen Bosphorus and its paleoclimatic implication based on a summary of the historical data	Book	The Black Sea Flood Question: Changes in Coastline, Climate, and Human Settlement, Publised by Springer		633-649

Table S3. Change in the river and lake ice cover duration.

	Period	Trend [days/100years]
Rivers		
Tulcea	1837 - 2016	-28.46***
Budapest	1775 - 2016	-19.62***
Nagymaros	1878 – 2016	-10.92***
Mohacs	1878 – 2016	-25.99***
Komarom	1876 – 2016	-7.65**
Angara	1775 – 1956	-1.31
NH TT	1880 - 2016	0.81***
Lakes		
Kallavesi	1834 – 2007	-15.74***
Oulujavri	1855 – 2006	-13.53***
Mjosa	1866 – 1997	-18.10***
Baikal	1869 – 2006	-16.62***
Balaton	1886 - 2017	-1.03

*** indicates 99% significance level

** indicates 95% significance level

References

1. van den Besselaar, E.J.M., Haylock, M.R., van der Schrier G. & Klein Tank A.M.G..A European Daily High-resolution Observational Gridded Data set of Sea Level Pressure*. J. Geophys. Res*. **116**, D11110 (2011).

2. Compo, G. P. & Co-authors. The Twentieth Century Reanalysis project. *Quart. J. Roy. Meteor. Soc*. **137**, 1–28 (2011).

3. Rayner, N. A. & Co-authors. Global analyses of sea surface temperature, sea ice, and night marine air temperature since the late nineteenth century. *J. Geophys. Res*. **108**, D14, 4407 (2003).

4. Luterbacher, J. et al. Reconstruction of sea level pressure fields over the Eastern North Atlantic and Europe back to 1500. *Climate Dynamics* **18**, 545 - 561 (2002).

5. Casty C., Raible C.C., Stocker T.F., Wanner H. & Luterbacher J. A European pattern climatology 1766–2000. *Climate Dynamics* 29: 791–805 (2007).

6. Takács, K. & Kern, Z. Long-term ice phenology records of Lake Balaton and the Danube River (East Central Europe). *PANGAEA*, https://doi.org/10.1594/PANGAEA.881056 (2017).

7. Hansen, J., Ruedy R., Sato M., & Lo K. Global surface temperature change, *Rev. Geophys.* **48**, RG4004, doi:10.1029/2010RG000345 (2010).

8. Reynolds, R. W. & Co-authors. Daily High-Resolution-Blended Analyses for Sea Surface Temperature. *J. Climate* 20, 5473-5496 (2007).

9. Dee, D. P. & Co-authors. The ERA-Interim reanalysis: configuration and performance of the data assimilation system. *Q.J.R. Meteorol. Soc*. 137, 553–597. doi: 10.1002/qj.828 (2011).

10. Taylor K.E., Stouffer R.J. & Meehl G.A. An overview of CMIP5 and the experiment design. *Bull Am Meteorol Soc* **93**(4), 485–498 (2012)
